# Machine-learning based MRI radiomics models for early detection of radiation-induced brain injury in nasopharyngeal carcinoma

**DOI:** 10.1186/s12885-020-06957-4

**Published:** 2020-06-01

**Authors:** Bin Zhang, Zhouyang Lian, Liming Zhong, Xiao Zhang, Yuhao Dong, Qiuying Chen, Lu Zhang, Xiaokai Mo, Wenhui Huang, Wei Yang, Shuixing Zhang

**Affiliations:** 1 Department of Radiology, The First Affiliated Hospital of Jinan University, Guangzhou, Guangdong China; 2 Jinan University, Guangzhou, Guangdong China; 3 Department of Radiology, Guangdong Provincial People’s Hospital/Guangdong Academy of Medical Sciences, Guangzhou, Guangdong China; 4 Guangdong Provincial Key Laboratory of Medical Image Processing, School of Biomedical Engineering, Southern Medical University, Guangzhou, China; 5 Zhuhai Precision Medical Center, Zhuhai People’s Hospital, Zhuhai Hospital Affiliated with Jinan University, Zhuhai, China; 6 Department of Catheterization Lab, Guangdong Cardiovascular Institute, Guangdong Provincial Key Laboratory of South China Structural Heart Disease, Guangdong Provincial People’s Hospital /Guangdong Academy of Medical Sciences, Guangzhou, Guangdong People’s Republic of China

**Keywords:** Radiation-induced temporal lobe injury, Nasopharyngeal carcinoma, Radiomics, Machine learning, Magnetic resonance imaging

## Abstract

**Background:**

Early radiation-induced temporal lobe injury (RTLI) diagnosis in nasopharyngeal carcinoma (NPC) is clinically challenging, and prediction models of RTLI are lacking. Hence, we aimed to develop radiomic models for early detection of RTLI.

**Methods:**

We retrospectively included a total of 242 NPC patients who underwent regular follow-up magnetic resonance imaging (MRI) examinations, including contrast-enhanced T1-weighted and T2-weighted imaging. For each MRI sequence, four non-texture and 10,320 texture features were extracted from medial temporal lobe, gray matter, and white matter, respectively. The relief and 0.632 + bootstrap algorithms were applied for initial and subsequent feature selection, respectively. Random forest method was used to construct the prediction model. Three models, 1, 2 and 3, were developed for predicting the results of the last three follow-up MRI scans at different times before RTLI onset, respectively. The area under the curve (AUC) was used to evaluate the performance of models.

**Results:**

Of the 242 patients, 171 (70.7%) were men, and the mean age of all the patients was 48.5 ± 10.4 years. The median follow-up and latency from radiotherapy until RTLI were 46 and 41 months, respectively. In the testing cohort, models 1, 2, and 3, with 20 texture features derived from the medial temporal lobe, yielded mean AUCs of 0.830 (95% CI: 0.823–0.837), 0.773 (95% CI: 0.763–0.782), and 0.716 (95% CI: 0.699–0.733), respectively.

**Conclusion:**

The three developed radiomic models can dynamically predict RTLI in advance, enabling early detection and allowing clinicians to take preventive measures to stop or slow down the deterioration of RTLI.

## Background

Radiotherapy remains the primary treatment modality for nasopharyngeal carcinoma (NPC) because of tumor’s anatomic location and radiosensitivity [1]. However, the medial temporal lobes are inevitably included in the target volume, which often results in brain injuries after several years [2]. Radiation-induced temporal lobe injury (RTLI) is thus a major neurological complication after radiotherapy, especially in patients with stage T3 or T4 disease [3].

According to the time of symptom occurrence post-radiotherapy, the course of RTLI can be divided into three stages: acute stage, subacute stage, and late stage [4, 5]. The acute injury occurs 2 weeks post-radiotherapy, which is normally reversible and resolved spontaneously. The subacute injury, occurs 1–6 months post-radiotherapy, with short-term symptoms and good prognosis. The late injury occurs > 6 months to several years post-radiotherapy, which is progressive and irreversible. Acute and subacute RTLI is silent, which can be latent for years until to the late stage.

Currently, the diagnosis of RTLI largely depends on magnetic resonance imaging (MRI) [6, 7]. However, its diagnostic value is limited because white matter edema and demyelinating performance generally reveal the disease in the late stage. Recently, functional imaging techniques such as dynamic contrast enhanced (DCE), diffusion-weighted imaging (DWI), magnetic resonance spectroscopy (MRS), diffusion tensor imaging (DTI) have been used to supply function and metabolism information to conventional MRI [8–11]. However, guaranteeing that the same voxel position is selected from the same patient for analysis each time is challenging, and the spatial resolution of tract-based spatial statistics limits the analysis of major white matter tracts and cannot reveal fine changes in the regional white matter structure. Therefore, new methods providing information for the RTLI at early stage are needed.

Radiomic approaches could potentially be applied as an effective solution. In general, this refers to the conversion of medical images into high-dimensional mineable data through high-throughput extraction of quantitative image features and subsequent data analysis, which goes beyond automating what can be done with the naked eye or imaging tools [12]. This is an emerging method for complex systems, especially for solid tumors [13]. A radiomic approach can help to process microstructural changes in the temporal lobe that are invisible to human eyes, thus enabling the prediction of RTLI, especially in the early stages. Predictive biomarkers of RTLI may enable the stratification of patients for customized treatment, and thus help to improve the quality of life and possibly prolong survival. Therefore, we aimed to develop and validate MRI radiomic biomarkers to dynamically predict RTLI in NPC patients after radiotherapy, and thereby enable clinicians to take preventive strategies to stop or slow down the deterioration of RTLI.

## Methods

### Longitudinal patient data

This retrospective, longitudinal cohort study was approved by the Ethics Committee of our institution, which waived the requirement for informed patient consent. Patients’ data were acquired from the institutional Picture Archiving and Communication System (PACS) between January 2006 and August 2016. A total of 242 NPC patients underwent radiotherapy were included. The inclusion criteria were as follows [14]: 1) patients with a pathologically proven NPC; 2) patients who were treated with three dimensional conformal radiotherapy (3D-CRT); 3) patients had regular follow-up by MRI according to the guidelines (every 3 months during the first year, every 6 months during the second year, and every 1 year during the following years); 4) patients with RTLI, for whom the diagnosis of RTLI was based on MRI; and 5) patients without RTLI, whose follow-up time was > 112 months. Patients with central nervous or other system diseases affecting the medial temporal lobe were excluded. Finally, 200 patients with RTLI and 42 patients without RTLI were included for analysis.

Demographic and pretreatment clinical characteristics were collected from PACS, including age, sex, overall stage, WHO type, radiation dose, and chemotherapy regimens. The accumulated radiation doses applied to the primary tumor were 66–76 Gy, delivered in 33–38 fractions. All the patients were treated with one fraction daily, 2 Gy per fraction, 5 days per week. During the study period, the institutional guidelines recommended no additional chemotherapy for patients with stage I–IIA disease, concurrent chemoradiotherapy for stage IIB disease, and concurrent chemoradiotherapy +/− introduced/ adjuvant chemotherapy for stages III–IVa.

### Diagnostic criteria for RTLI

The diagnostic criteria for RTLI were as follows: (i) white matter lesions (finger-like lesions of increased signal intensity in T2-weighted (T2-w) images); (ii) contrast-enhanced lesions (lesions with or without necrosis in contrast-enhanced T1-weighted (CET1-w) images with heterogeneous signal abnormalities in T2-w images); and (iii) cysts (round or oval-shaped well-defined lesions of very high signal intensity in T2-w images with thin or imperceptible walls). Differential diagnosis was performed to ensure that the changes were not due to other factors, such as tumor recurrence. The latency of RTLI was measured from the day of radiotherapy completion to the date of MRI diagnosis. After completing radiotherapy, all the patients underwent regular follow-up during the latent period, as described in the inclusion criteria. Nasopharyngeal mirror, endoscopic, physical examinations, as well as MRI, were performed during the follow-up period. All MRI images were retrospectively reviewed by two independent radiologists (with 10 and 20 years of experience in NPC, respectively), and disagreements were resolved by consensus.

### MRI acquisition

All the patients underwent 1.5 T MRI examination (GE Signa Excite HD twinSpeed). The MRI sequences included T1-w spin-echo images (TR/TE: 500/8 ms, FOV = 22 × 22 cm, NEX = 2.0, slice thickness = 4 mm, interslice gap = 0.8 mm), axial T2-w spin-echo images (TR/TE: 5000/8 ms, FOV = 22 × 22 cm, NEX = 2.0, slice thickness = 4 mm, spacing = 1.0 mm, interslice gap = 0.8 mm), and axial CET1-w spin-echo images (TR/TE: 500/8 ms, FOV = 22 × 22 cm, NEX = 2.0, slice thickness = 4 mm, interslice gap = 0.8 mm). Bolus injection of contract agent (0.1 mmol/kg body weight; Magnevist, Schering, Berlin, Germany) was conducted before CET1-w imaging.

### Radiomic pipeline

**Fig. 1:**
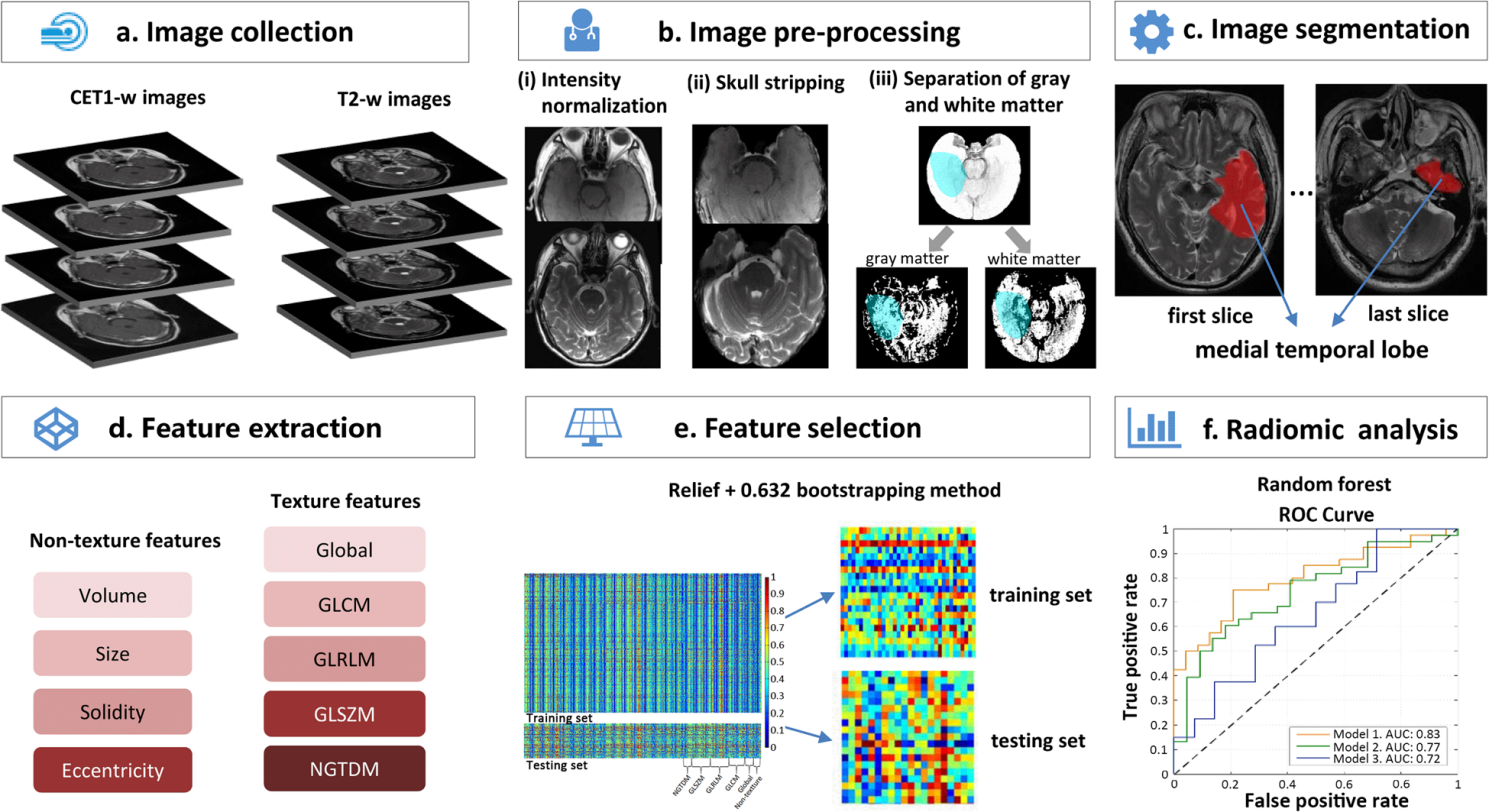
The radiomic process mainly involves **a**) MR images acquired; **b**) MR image pre-processing, including (i) intensity normalization, (ii) skull stripping, and (iii) gray /− white matter separation from the medial temporal lobe; **c**) medial temporal lobe segmentation; **d**) radiomic feature extraction; **e**) radiomic feature selection; and **f**) radiomic analysis

The radiomic process mainly comprises: a) MRI image acquisition; b) image pre-processing, including intensity normalization, skull stripping, and gray/white matter separation from medial temporal lobe; c) medial temporal lobe segmentation; d) feature extraction; e) feature selection; and f) radiomic analysis (Fig. 1).

### MRI data pre-processing

Considering the heterogeneity of the intra- and inter-patient MRI images, pre-processing is essential. All MRI images were processed by applying the typical procedures, including bias correction, intensity normalization, skull stripping, and segmentation of different tissue types. Firstly, the N4 Integration Tool Kit (ITK) MRI bias correction algorithm was used to remove the bias field artifacts. Secondly, intensity normalization was performed to reduce the variance across the MR images acquired by different manufacturers from different patients, and during different follow-up examinations. It was separately conducted for each of the different follow-up time points. The process consisted of two steps. In the first step, the parameters of a histogram transformation were learned from the input images and a few additional input parameters were determined. In the second step (transformation), the images were transformed using the parameters learned in the first step. This transformation is image dependent and needs to be done for each given image. Thirdly, MR brain images were segmented automatically into brain and non-brain regions using the Brain Extraction Tool (BET) in FSL package [15–17] by employing the BET command to generate a brain mask and applying a set of locally adaptive model forces. Then, non-brain tissue was removed from the anatomical MR images. The Automated Segmentation Tool developed by FMRIB was then employed, which can segment 3D brain images into different tissue types, and correct spatial intensity variations (also known as bias fields or radio-frequency inhomogeneities). The medial temporal lobe, gray matter, and white matter were segmented automatically. The underlying method was based on a hidden Markov random field model and an associated expectation-maximization algorithm. The whole process is fully automated, which is robust and reliable, and can produce bias-field-corrected input images and probabilistic and/or partial volume tissue segmentation.

### Medial temporal lobe segmentation

We used the ITK-SNAP (open source software; www.itk-snap.org) software for 3D manual segmentation. The region of interest (ROI) covered the middle and lower portions of the medial temporal lobe, from the slice of the cerebral peduncle to the last slice of the medial temporal lobe in the axial CET1-w and T2-w images. The ROI was delineated in the unilateral and left medial temporal lobes if the RTLI involved the unilateral and bilateral medial temporal lobes, respectively. All manual segmentations were performed by a radiologist with 10 years of experience and validated by a senior radiologist with 20 years of experience (largely with NPC).

### Radiomic feature extraction and selection

Feature extraction was performed using MATLAB 2014a (MathWorks, Natick, MA, USA) and based on CET1-w and T2-w images. Four non-texture and 43 types of texture features were provided. In the extraction of texture features, we used three types of texture parameter including five ratios of wavelet band-pass filtering, six scale values of Isotropic voxel size and two quantization algorithms, and 4 gray levels. Thus, for each MRI sequence, four non-texture and 10,320 (43*5*6*2*4) texture features can be extracted from medial temporal lobe, gray matter and white matter, respectively. The radiomic feature extraction methods were reported in the Appendix A1.

The extracted abundant information from the medial temporal lobe, gray matter, white matter of CET1-w and T2-w images was not suitable for direct modeling because not all features were effective for the detection of RTLI. Therefore, the feature selection for the prediction model was performed using the relief algorithm proposed by Kononenko et al. [18], in which attributes are estimated according to how well their values are distinguished among instances that are close to each other. The features were selected by arranging the weights in descending order. For each set of texture parameters, we obtained two top features; hence, 480 (2*5*6*2*4) features with high expression low redundancy were selected from the four non-texture and 10,320 texture features for CET1-w, T2-w, and the combined CET1-w and T2-w images. The whole dataset was randomly divided into the training cohort (*n* = 80%) and the testing cohort (*n* = 20%). 80% patients were resampled 1000 times using the 0.632 bootstrapping method to generate 1000 different training and validation subsets. Then, the 480 features was used to build the model by random forest method with 1000 different training subset and validation subset results. The feature selection method is demonstrated as follows:

The reverse time series of the latency imaging data for our patient cohort can be defined as the matrix X = {x_ij_ : i = 1, 2, …, M; j = t_1_, t_2_, …, t_N_}, where M is the number of patients, *j* represents different time series, and N is the last follow-up scan for the RTLI-positive group and the last recorded scan for the RTLI-negative group. We used the last three follow up MRI scans (*N*-3, *N*-2, and *N*-1) for RTLI prediction. The bootstrap samples were ${\mathrm{X}}^{\ast }=\left\{{\mathrm{x}}_{\mathrm{ij}}^{\ast }:\mathrm{i}=1,2,\dots, \mathrm{M};\mathrm{j}={\mathrm{t}}_1,{\mathrm{t}}_2,\dots, {\mathrm{t}}_{\mathrm{N}}\right\}$. The bootstrap sample of *x*_*i*_ randomly drawn input variables, which replaced the available sample X for each time series. The set of original data vectors not appearing in X* is denoted as X*(0). The feature selection was then conducted by imbalance-adjusted bootstrap resampling (1000 times). The prediction performance was evaluated by the 0.632 + bootstrap AUC:

$$ {\displaystyle \begin{array}{c}{\left[\mathrm{A}\overset{\frown }{\mathrm{U}}\mathrm{C}\right]}_{0.632+}=\frac{1}{\mathrm{B}}\sum \limits_{\mathrm{b}=1}^{\mathrm{B}}\left[\left(1\hbox{-} \mathrm{a}\left(\mathrm{b}\right)\right)\cdot \mathrm{AUC}\left(\mathrm{X},\mathrm{X}\right)+\mathrm{a}\left(\mathrm{b}\right)\cdot \mathrm{AUC}\hbox{'}\left({\mathrm{X}}^{\ast \mathrm{b}},{\mathrm{X}}^{\ast \mathrm{b}}(0)\right)\right]\kern6.5em \\ {}\mathrm{where}\kern0.5em \mathrm{AUC}\hbox{'}\left({\mathrm{X}}^{\ast \mathrm{b}},{\mathrm{X}}^{\ast \mathrm{b}}(0)\right)=\max \left\{0.5,\mathrm{AUC}\left({\mathrm{X}}^{\ast \mathrm{b}},{\mathrm{X}}^{\ast \mathrm{b}}(0)\right)\right\},\kern0.5em \mathrm{a}\left(\mathrm{b}\right)=\frac{0.632}{1\hbox{-} 0.368\cdot \mathrm{R}\left(\mathrm{b}\right)}\\ {}\mathrm{a}\mathrm{nd}\kern0.5em \mathrm{R}\left(\mathrm{b}\right)=\left\{\frac{\begin{array}{c}1\kern12em \\ {}\mathrm{AUC}\left(\mathrm{X},\mathrm{X}\right)\hbox{-} \mathrm{AUC}\left({\mathrm{X}}^{\ast \mathrm{b}},{\mathrm{X}}^{\ast \mathrm{b}}(0)\right)\end{array}}{\begin{array}{c}\mathrm{AUC}\left(\mathrm{X},\mathrm{X}\right)\hbox{-} 0.5\\ {}0\kern12em \end{array}}\begin{array}{c}\mathrm{if}\kern0.5em \mathrm{AUC}\left({\mathrm{X}}^{\ast \mathrm{b}},{\mathrm{X}}^{\ast \mathrm{b}}(0)\right)\le 0.5\kern2em \\ {}\mathrm{if}\kern0.5em 2>\frac{\mathrm{AUC}\left(\mathrm{X},\mathrm{X}\right)}{\mathrm{AUC}\left({\mathrm{X}}^{\ast \mathrm{b}},{\mathrm{X}}^{\ast \mathrm{b}}(0)\right)}>1\\ {}\mathrm{otherwise}\kern12em \end{array}\right\}\kern2.5em \end{array}} $$

Finally, we selected 20 top ranking features from the 480 features for further modeling.

### Three prediction models

**Fig. 2:**
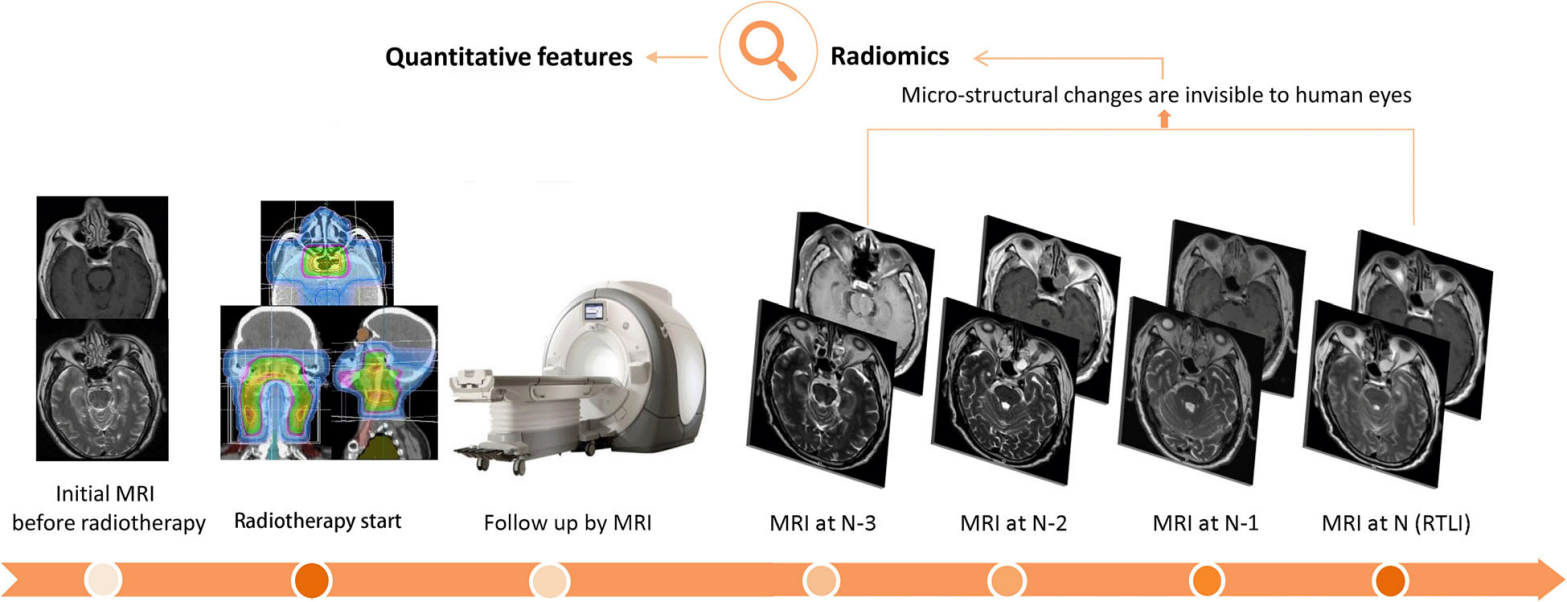
All NPC patients underwent pre-treatment MR scans and then received radiotherapy. After radiotherapy, they underwent regular MRI follow-up to monitoring the treatment response. We developed three radiomic models, models 1, 2, and 3, to predict RTLI at the last 1, 2, and 3 MRI scans (N-1, N-2, and N-3) before MRI confirmation (defined as N) respectively

We established three radiomic models—models 1, 2, and 3—to predict RTLI at N-1, N-2, and N-3 follow up MRI scans in different times before RTLI confirmation on MRI scan, respectively (Fig. 2). The prediction models were developed by random forest method and then validated in the remaining 20% patients. The description of random forest is shown in the Appendix A2. To reduce the imbalance between RTLI-positive and RTLI-negative samples, the last three follow up MR scans of the RTLI-negative (*n* = 42) patients were used in the three models. Appendix A3 presents the number of MRI scans of RTLI-positive and RTLI-negative patients in the models 1, 2, and 3. For each model, we tried different sets of top-ranking features (*n* = 1, 5, 10, 15, and 20). We then compared the predictive performance of the models based on different combinations of segmented tissues (medial temporal lobe, gray matter, and white matter), MRI sequences (CET1-w, T2-w and combined CET1-w and T2-w), and number of top ranking features (1/5/10/15/20).

### Statistical analysis

SPSS 23.0 (IBM, Armonk, NY), and MATLAB 2014a (Mathwork-Natick, MA) software were used for the statistical analyses. Continuous variables were expressed as mean plus or minus standard deviation (SD), while categorial variables were expressed as counts and percentages. The codes for this radiomic study was available at https://github.com/mvallieres/radiomics. The predictive performance of the models was assessed by measuring the AUC. The average AUC of radiomic models was obtained by bootstrapping for 1000 times. A *P* < 0.05 was considered as statistically significant.

## Results

This retrospective study included 242 patients (171 men and 71 women; mean age 48.5 ± 10.4 years). The median follow-up time and latency from 3D-CRT until RTLI were 46 months (interquartile range, 33–69 months) and 41 months (interquartile range 30–52 months), respectively. The longest latency was 112 months. In total, 105 and 95 patients had unilateral and bilateral RTLI, respectively. Of the RTLI cases, 12 (6%) and 188 (94%) were at stage T1–2 and T3–4, respectively. The patient characteristics are summarized in Table 1.

**Table 1:** Basic characteristics of 242 patients

Characteristics	
Sex	
Male	171 (70.7%)
Female	71 (29.3%)
Age (years)	48.5 ± 10.4
Overall stage	
I	7 (2.9%)
II	7 (2.9%)
III	92 (38.0%)
IV	136 (56.2%)
WHO type	
I	0 (0%)
II	23 (9.5%)
III	219 (90.5%)
Latency (median, months)	41
Radiation dose (Gy)	32 ± 5.39
Chemotherapy	
Yes	233 (96.3%)
No	9 (3.7%)

### Radiomic feature extraction and selection

For separate CET1-w and T2-w images, four non-texture and 10,320 texture features were extracted from medial temporal lobe, gray matter, and white matter, respectively. A total of 480 features were retained after initially selected by relief method for CET1-w, T2-w, and the combined CET1-w and T2-w images, respectively. Twenty top features were selected by 0.632 + bootstrap AUC from the 480 features. The results showed all 20 top features were texture features.

### Predictive performance of machine-learning-based radiomic models

Models 1, 2, 3 with non-texture features achieved AUCs of 0.680 (95% confidence interval [CI] 0.672–0.688), 0.550 (95% CI: 0.544–0.556), and 0.550 (95% CI: 0.546–0.554), respectively.

The 20 top texture features for each model were compared by their AUCs (Appendix A4). The features derived from T2-w images achieved higher performance than those extracted from CET1-w images.

**Fig. 3:**
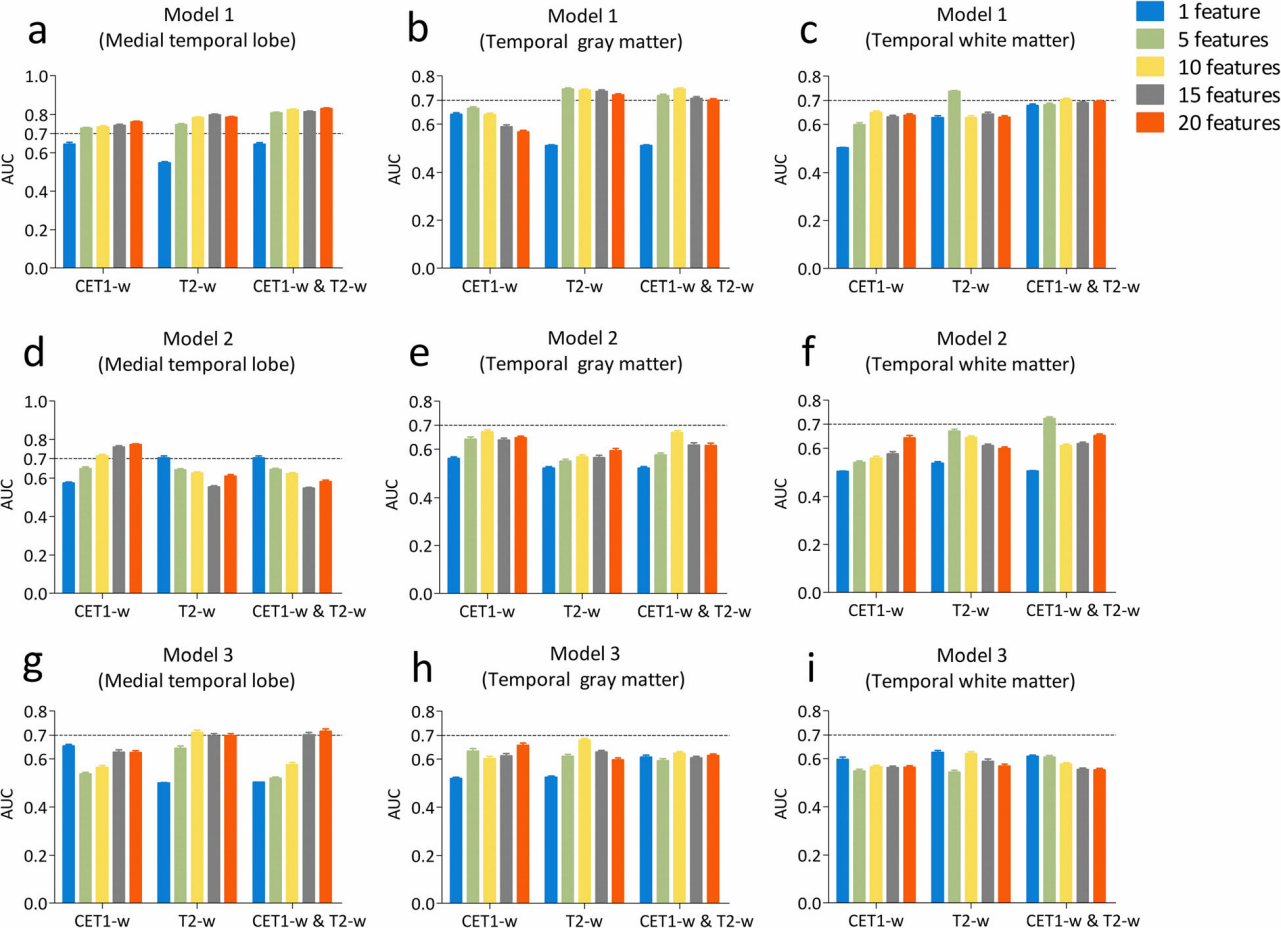
Comparison of AUCs among three prediction models using different combinations of radiomic features (*n* = 1, 5, 10, 15 and 20) in the testing cohort. **a**-**c** model 1 using features derived from medial temporal lobe, gray matter, and white matter respectively. **d**-**f** model 2 using features derived from medial temporal lobe, gray matter, and white matter respectively. **g**-**i** model 3 using features derived from medial temporal lobe, gray matter, and white matter respectively

In the training cohort, models 1, 2, and 3, with all 20 top features derived from combined CET1-w (*n* = 2) and T2-w (*n* = 18) images, CET1-w images only, and combined CET1-w (*n* = 9) and T2-w (*n* = 11) images of medial temporal lobe, yielded AUCs of 0.851 (95% CI: 0.841–0.861), 0.738 (95% CI: 0.721–0.755), and 0.777 (95% CI: 0.754–0.801), respectively (Appendix A5). In the testing cohort, models 1, 2, and 3, yielded AUCs of 0.830 (95% CI: 0.823–0.837), 0.773 (95% CI: 0.763–0.782), and 0.716 (95% CI: 0.699–0.733), respectively (Fig. 3).

## Discussion

This is the first study on the prediction of brain injuries due to radiation using MRI radiomic technology. On the basis of the MRI data collected from 242 NPC patients with or without RTLI, we developed three prediction models combining the 20 MRI radiomic features most significantly associated with early RTLI. The radiomic models with longitudinal MRI yielded AUCs of 0.872 (95% CI: 0.862–0.881), 0.836 (95% CI: 0.823–0.849), and 0.780 (95% CI: 0.759–0.800) for RTLI prediction in advance.

Conventional MR imaging can only be used to evaluate morphologic changes of late radiation injury to the temporal lobes. However, structural and functional MR imaging biomarker that is sensitive to early irradiation brain injury has been previously detected [19]. Leng X et al. analyzed the microstructural dynamic alterations in all brain lobes after radiotherapy in NPC patients at different times, by using DTI for white matter and voxel-based morphometry for gray matter volume [20]. Fractional anisotropy values of whiter matter and gray matter volume decreased markedly at acute and subacute stages after radiotherapy [21–23]. Lin J et al. observed increased cortical thickness in NPC patients in the early period after radiotherapy [24]. Diffusion kurtosis imaging (DKI) can detect the early presence of relatively subtle radiation-induced changes before temporal lobe necrosis [25, 26]. Altered brain functional connections were significantly correlated to the Montreal Cognitive Assessment scores in NPC patients, which may serve as a potential biomarker of the brain functional impairments [27, 28]. Thus, structural and functional MRI were more sensitive than conventional MRI in determining radiation induced brain damage.

A radiomic approach enables the identification of imaging phenotypes and can reflect pathophysiological changes. The three radiomic models using T2-w images demonstrated better predictive performance than those using CET1-w images. This may be because T2-w images can assist the better detection of white matter lesions, and a homogeneous increase in T2-w signal intensity of a white matter lesion is believed to represent demyelination, gliosis, and edema [6]. Preclinical studies have suggested that white matter lesions are the earliest form of radiation injury [29, 30]. White matter is more sensitive to radiotherapy than gray matter because it has a richer vascular supply. Although white matter lesions are universal and the first MRI manifestation, the mechanisms of radiation damage are complex, and no universally accepted argument has been established. Cerebrovascular injury and remodeling is an underlying hypothesis for the development of RTLI. Recently, a genome-wide association study implicated the genetic susceptibility gene CEP128 in RTLI development [31]. Generally, the particular mechanism of RTLI includes not only demyelination, softening, and necrosis of white matter, but also nerve and glial cell damage caused by direct radiation, which are related to gray matter [32]. In some cases, gray matter damage could be the only abnormal change in the MR images of RTLI patients. Therefore, radiomic features extracted from medial temporal lobe have higher predictive performance than those from white or gray matter alone. CET1-w images reflect heterogeneity and architecture which are related to radiation necrosis in a histology RTLI analysis. The close relationship between contrast enhancement and radiation necrosis is well recognized, and focal disruption of the blood–brain barrier has been reported to correspond with necrosis [33]. Radiation can lead to hyaline degeneration in the blood vessel wall, intimal reactive hyperplasia, and increased vascular permeability [34], which can be better indicated by CET1-w images. Our results suggested that the different MRI measures contain complementary information. A combination of these measures may therefore improve the predictive performance of RTLI.

Our RTLI prediction models would improve patient management as well as clinicians’ decision-making in clinical practice. A complete head and neck examination should be performed every 12 months for NPC patients diagnosed more than 5 years previously [35], but for those patients at high risk for RTLI, this time interval is too long for detection: 6 months or less is recommended. Additionally, the follow-up examinations of these patients should include brain MRI scans instead of only nasopharyngeal MRI scans to avoid misdetection of RTLI. For patients with early RTLI, some mitigators and therapeutics may be effective in preventing or ameliorating RTLI [36–38]. Note that neuroprotective treatments have to be given before, during, and continuously after irradiation.

This study also has some limitations. Firstly, the number of RTLI-negative patients was small because they were required to have been followed for at least 112 months, and it was difficult to include such patients. Secondly, NPC patients received 3D-CRT instead of intensity modulated radiotherapy (IMRT) because our institution didn’t use IMRT during the follow-up period. Finally, this is a single institution study and may not be transferable to other institutions. Therefore, more prospective studies are warranted to validate the performance of our prediction models.

## Conclusions

We developed three non-invasive models by combining radiomic features extracted from MR images of the medial temporal lobe to predict RTLI dynamically in advance. These prediction tools provide the basis for decisions regarding the early detection and preventive therapy of RTLI. Furthermore, the present study provides valuable insights into the application of radiomics in tumor radiotherapy related complications for the first time, thereby enhancing the capacity of radiomics. However, multi-center retrospective validation studies and prospective randomized clinical trials should be performed to obtain high-level evidences for future clinical applications and investigate the generalization of the prediction models to other cancers. Moreover, we call for standardization of future radiomic studies to make sure that they are generalized and can be transferred to other institutions.

## Supplementary information

**Additional file 1: Appendix A1.** radiomic feature extraction methodology. **Appendix A2.** The description of random forest method. **Appendix A3.** Table 1. MRI examinations of RTLI-positive and RTLI-negative patients in models 1, 2 and 3. **Appendix A4.** Average AUC of selected radiomic features for three models. **Appendix A5.** Figure 1. Comparison of AUCs between three prediction models (models 1, 2, and 3) using different combinations of radiomic features (*n* = 1, 5, 10, 15 and 20) in the training cohort. (a-c) model 1 using features derived from the medial temporal lobe, temporal gray matter, and temporal white matter respectively. (d-f) model 2 using features derived from the medial temporal lobe, temporal gray matter, and temporal white matter respectively. (g-i) model 3 using features derived from the medial temporal lobe, temporal gray matter, and temporal white matter respectively.

## Data Availability

The datasets used and/or analysed during the current study available from the corresponding author on reasonable request.

## Abbreviations

RTLI: Radiation-induced temporal lobe injury
NPC: Nasopharyngeal carcinoma
MRI: Magnetic resonance imaging
CET1-w: Contrast-enhanced T1-weighted
T2-w: T2-weighted
AUC: Area under the curve
3D-CRT: Three dimensional conformal radiotherapy
