# Supplementary material for: Machine-learning based MRI radiomics models for early detection of radiation-induced brain injury in nasopharyngeal carcinoma
Source: BMC Cancer. 2020 Jun 1;20:502. doi: 10.1186/s12885-020-06957-4 (PMC7268644; doi:10.1186/s12885-020-06957-4)

**Supplementary Materials**

**Appendix A1: radiomic feature extraction methodology**

***Non-texture features*.** Four non-texture radiomic features were extracted for completeness.

1) **Volume:** Number of voxels in the medial temporal lobe region times the voxel dimensions.

2) **Size:** Longest diameter of the medial temporal lobe region.

3) **Solidity:** Ratio of the number of voxels in the medial temporal lobe region to the number of voxels in the 3D convex hull of the medial temporal lobe region (smallest polyhedron containing the temporal lobe region).

4) **Eccentricity:** The best-fit ellipsoid was obtained for the medial temporal lobe region, with an eccentricity given by$\sqrt{1-a\times b/c^{2}}$, where *c*, *a*, and *b* are the longest, second-longest, and third-longest semi-principal axes of the ellipsoid respectively.

***Texture features*:** Forty-three texture features were obtained (Supplementary Table 1). The texture extraction parameters were as follows. The influences of the following three extraction parameters on the predictive texture values were investigated.

1) **Wavelet band-pass filtering:** The parameters were set based on the wavelet properties. Different weights were applied to the band-pass sub-bands of the targeted region as compared to the low- and high-frequency sub-bands. Ratios of 1/2, 2/3, 1, 3/2, and 2 were tested.

2) **Isotropic voxel size:** All volumes were resampled to an isotropic voxel size set to a desired resolution using cubic interpolation. Scale values of 1, 2, 3, 4, and 5 mm and initial in-plane resolution were tested.

3) **Gray level quantization:** The full intensity range of the temporal lobe region was quantized to a small number of gray levels. There are two related extraction parameters:

a. Quantization (Q) algorithm (equal-probability and Lloyd-max);

b. Number of gray levels (Ng) in the quantized volume; values of 8, 16, 32, and 64 were tested.

Global: Global features were extracted from the intensity histogram of the ROI. Let: $p_{i}$be the gray level probability with gray level$i$, $x_{i}$be the number of voxels, $\mu$be the average value, and $\sigma$be the standard deviation.

**Variance:**

$$v\mathrm{ar}iance=\sum_{i=1}^{n} p_{i}\left(x_{i}-\mu\right)^{2}$$

**Skewness:**

$$skewness=\frac{\sum\left(x_{i}-\mu\right)^{3}}{n\sigma^{3}}$$

**Kurtosis:**

$$kurtosis=\frac{\sum\left(x_{i}-\mu\right)^{4}}{n\sigma^{4}}$$

**Gray-level co-occurrence matrix (GLCM):** GLCMs are calculated in 13 directions of 3D spaces. Let: $p(i,j)$be the co-occurrence matrix for an arbitrary $\delta$and $\theta$, $p(i,j)$be the normalized co-occurrence matrix, $N_{g}$be the number of discrete intensity levels in the image, $p_{x}(i)$be the marginal row probabilities, $p_{y}(j)$be the marginal column probabilities, $\mu_{x}$be the mean gray level intensity of $p_{x}$, $\mu_{y}$be the mean gray level intensity of $p_{y}$, $\sigma_{x}$be the standard deviation of $p_{x}$, and $\sigma_{y}$be the standard deviation of $p_{y}$.

$$p(i,j)=\frac{P(i,j)}{\sum P(i,j)},\text{ }p_{x}(i)=\sum_{j=1}^{N_{g}} P(i,j),\text{ }p_{y}(j)=\sum_{i=1}^{N_{g}} P(i,j)$$

$$\mu_{x}=\sum_{i=1}^{N_{g}} p_{x}(i)i,\text{ }\mu_{y}=\sum_{j=1}^{N_{g}} p_{y}(j)j$$

$$\begin{matrix} p_{x+y}(k)=\sum_{i=1}^{N_{g}} \sum_{j=1}^{N_{g}} p(i,j),\text{ }where\text{ }i+j=k,\text{ }and\text{ }k=2,3,\ldots,2N_{g} \\ p_{x-y}(k)=\sum_{i=1}^{N_{g}} \sum_{j=1}^{N_{g}} p(i,j),\text{ }where\text{ }\left|i-j\right|=k,\text{ }and\text{ }k=0,1,\ldots,N_{g}-1 \end{matrix}$$

$$\begin{matrix} HX=-\sum_{i=1}^{N_{g}} p_{x}(i)\log_{2}(p_{x}(i)),\text{ }HY=-\sum_{j=1}^{N_{g}} p_{y}(j)\log_{2}(p_{y}(i)),\text{ }HXY=-\sum_{i=1}^{N_{g}} \sum_{j=1}^{N_{g}} p(i,j)\log_{2}(p(i,j)) \\ HXY1=-\sum_{i=1}^{N_{g}} \sum_{j=1}^{N_{g}} p(i,j)\log_{2}(p_{x}(i)p_{y}(j)),\text{ }HXY2=-\sum_{i=1}^{N_{g}} \sum_{j=1}^{N_{g}} p_{x}(i)p_{y}(j)\log_{2}(p_{x}(i)p_{y}(j)) \end{matrix}$$

**Energy:**

$$\text{energy}=\sum_{i=1}^{N_{g}} \sum_{j=1}^{N_{g}} [p\left(i,j\right)]^{2}$$

**Contrast:**

$$contrast=\sum_{i=1}^{N_{g}} \sum_{j=1}^{N_{g}} \left(i-j\right)^{2}p\left(i,j\right)$$

**Entropy:**

$$Entropy=-\sum_{i=1}^{N_{g}} \sum_{j=1}^{N_{g}} p\left(i-j\right)\log_{2}[p\left(i,j\right)]$$

**Homogeneity:**

$$\text{Homogeneity}=\sum_{i=1}^{N_{g}} \sum_{j=1}^{N_{g}} \frac{p\left(i,j\right)}{1+\left|i-j\right|}$$

**Correlation:**

$$correlation=\frac{\sum_{i=1}^{N_{g}} \sum_{j=1}^{N_{g}} p\left(i,j\right)ij-\mu_{x}\mu_{y}}{\sigma_{x}(i)\sigma_{y}(j)}$$

**Sum Average:**

$$Sum\text{ }average=\frac{1}{N_{g}\times N_{g}}\sum_{i=1}^{N_{g}} \sum_{j=1}^{N_{g}} \left[p\left(i,j\right)i+p\left(i,j\right)j\right]$$

**Variance:**

$$\mathrm{var}iance=\frac{1}{N_{g}\times N_{g}}\sum_{i=1}^{N_{g}} \sum_{j=1}^{N_{g}} \left[\left(i-\mu_{x}\right)^{2}p\left(i,j\right)+\left(j-\mu_{y}\right)^{2}p\left(i,j\right)\right]$$

**Dissimilarity:**

$$Dissimilarity=\sum_{i=1}^{N_{g}} \sum_{j=1}^{N_{g}} \left|i-j\right|p(i,j)$$

**Auto Correlation:**

$$Auto\text{ }Correlation=\sum_{i=1}^{N_{g}} \sum_{j=1}^{N_{g}} p\left(i,j\right)ij$$

**Gray-level run-length matrix (GLRLM):** GLRLMs quantify gray level runs which are defined as the length in the number of pixels. Let: $P(i,j|\theta)$be the run length matrix for an arbitrary direction $\theta$, $P(i,j|\theta)$be the normalized run length matrix, $N_{g}$be the number of discreet intensity values in the image, $N_{\tau}$be the number of discreet run lengths in the image, $N_{p}$be the number of voxels in the image, and $N_{z}(\theta)$be the number of runs in the image along angle $\theta$.

$$N_{z}\left(\theta\right)=\sum_{i=1}^{N_{g}} \sum_{j=1}^{N_{\tau}} P\left(i,j\left|\theta\right.\right),\text{ }where\text{ }1\leq N_{z}\left(\theta\right)\leq N_{P}$$

$$P\left(i,j\left|\theta\right.\right)=\frac{P\left(i,j\left|\theta\right.\right)}{N_{z}\left(\theta\right)}$$

**Short Run Emphasis (SRE):**

$$SRE=\frac{\sum_{i=1}^{N_{g}} \sum_{j=1}^{N_{\tau}} \frac{P\left(i,j\left|\theta\right.\right)}{j^{2}}}{N_{z}\left(\theta\right)}$$

**Long Run Emphasis (LRE):**

$$LRE=\frac{\sum_{i=1}^{N_{g}} \sum_{j=1}^{N_{\tau}} P\left(i,j\left|\theta\right.\right)j^{2}}{N_{z}\left(\theta\right)}$$

**Gray-Level Non-uniformity (GLN):**

$$GLN=\frac{\sum_{i=1}^{N_{g}} \left(\sum_{j=1}^{N_{\tau}} P\left(i,j\left|\theta\right.\right)\right)^{2}}{N_{z}\left(\theta\right)}$$

**Run-Length Non-uniformity (RLN):**

$$RLN=\frac{\sum_{j=1}^{N_{\tau}} \left(\sum_{i=1}^{N_{g}} P\left(i,j\left|\theta\right.\right)\right)^{2}}{N_{z}\left(\theta\right)}$$

**Run Percentage (RP):**

$$RP=\frac{N_{z}\left(\theta\right)}{N_{p}}$$

**Low Gray-Level Run Emphasis (LGRE):**

$$LGRE=\frac{\sum_{i=1}^{N_{g}} \sum_{j=1}^{N_{\tau}} \frac{P\left(i,j\left|\theta\right.\right)}{i^{2}}}{N_{z}\left(\theta\right)}$$

**High Gray-Level Run Emphasis (HGRE):**

$$HGRE=\frac{\sum_{i=1}^{N_{g}} \sum_{j=1}^{N_{\tau}} P\left(i,j\left|\theta\right.\right)i^{2}}{N_{z}\left(\theta\right)}$$

**Short Run Low Gray-Level Emphasis (SRLGE):**

$$SRLGE=\frac{\sum_{i=1}^{N_{g}} \sum_{j=1}^{N_{\tau}} \frac{P\left(i,j\left|\theta\right.\right)}{i^{2}j^{2}}}{N_{z}\left(\theta\right)}$$

**Short Run High Gray-Level Emphasis (SRHGE):**

$$SRHGE=\frac{\sum_{i=1}^{N_{g}} \sum_{j=1}^{N_{\tau}} \frac{P\left(i,j\left|\theta\right.\right)i^{2}}{j^{2}}}{N_{z}\left(\theta\right)}$$

**Long Run Low Gray-Level Emphasis (LRLGE):**

$$LRLGE=\frac{\sum_{i=1}^{N_{g}} \sum_{j=1}^{N_{\tau}} \frac{P\left(i,j\left|\theta\right.\right)j^{2}}{i^{2}}}{N_{z}\left(\theta\right)}$$

**Long Run High Gray-Level Emphasis (LRHGE):**

$$LRHGE=\frac{\sum_{i=1}^{N_{g}} \sum_{j=1}^{N_{\tau}} P\left(i,j\left|\theta\right.\right)i^{2}j^{2}}{N_{z}\left(\theta\right)}$$

**Gray-Level Variance (GLV):**

$$GLV=\sum_{i=1}^{N_{g}} \sum_{j=1}^{N_{\tau}} p\left(i,j\left|\theta\right.\right)\left(i-\sum_{i=1}^{N_{g}} \sum_{j=1}^{N_{\tau}} p\left(i,j\left|\theta\right.\right)i\right)^{2}$$

**Run-Length Variance (RLV):**

$$RLV=\sum_{i=1}^{N_{g}} \sum_{j=1}^{N_{\tau}} p\left(i,j\left|\theta\right.\right)\left(j-\sum_{i=1}^{N_{g}} \sum_{j=1}^{N_{\tau}} p\left(i,j\left|\theta\right.\right)j\right)^{2}$$

**Gray-level size zone matrix (GLSZM):** GLSZMs quantify gray level zones in an image. Let: $P(i,j)$be the size zone matrix, $P(i,j)$be the normalized size zone matrix, $N_{g}$be the number of discreet intensity values in the image, $N_{s}$be the number of discreet zone sizes in the image, $N_{p}$be the number of voxels in the image, and $N_{z}$be the number of zones in the ROI.

$$N_{z}=\sum_{i=1}^{N_{g}} \sum_{j=1}^{N_{s}} P\left(i,j\right),where1\leq N_{z}\leq N_{p}$$

$$\text{ }P\left(i,j\right)=\frac{P\left(i,j\right)}{N_{z}}$$

**Small Zone Emphasis (SZE):**

$$SZE=\frac{\sum_{i=1}^{N_{g}} \sum_{j=1}^{N_{s}} \frac{P\left(i,j\right)}{j^{2}}}{N_{z}}$$

**Large Zone Emphasis (LZE):**

$$LZE=\frac{\sum_{i=1}^{N_{g}} \sum_{j=1}^{N_{s}} P\left(i,j\right)j^{2}}{N_{z}}$$

**Gray-Level Non-uniformity (GLN):**

$$GLN=\frac{\sum_{i=1}^{N_{g}} \left(\sum_{j=1}^{N_{s}} P\left(i,j\right)\right)^{2}}{N_{z}}$$

**Zone-Size Non-uniformity (ZSN):**

$$ZSN=\frac{\sum_{j=1}^{N_{s}} \left(\sum_{i=1}^{N_{g}} P\left(i,j\right)\right)^{2}}{N_{z}}$$

**Zone Percentage (ZP):**

$$ZP=\frac{N_{z}}{N_{p}}$$

**Low Gray-Level Zone Emphasis (LGZE):**

$$LGZE=\frac{\sum_{i=1}^{N_{g}} \sum_{j=1}^{N_{s}} \frac{P\left(i,j\right)}{i^{2}}}{N_{z}}$$

**High Gray-Level Zone Emphasis (HGZE):**

$$HGZE=\frac{\sum_{i=1}^{N_{g}} \sum_{j=1}^{N_{s}} P\left(i,j\right)i^{2}}{N_{z}}$$

**Small Zone Low Gray-Level Emphasis (SZLGE):**

$$SZLGE\text{=}\frac{\sum_{i=1}^{N_{g}} \sum_{j=1}^{N_{s}} \frac{P\left(i,j\right)}{i^{2}j^{2}}}{N_{z}}$$

**Small Zone High Gray-Level Emphasis (SZHGE):**

$$SZHGE=\frac{\sum_{i=1}^{N_{g}} \sum_{j=1}^{N_{s}} \frac{P\left(i,j\right)i^{2}}{j^{2}}}{N_{z}}$$

**Large Zone Low Gray-Level Emphasis (LZLGE):**

$$LZLGE=\frac{\sum_{i=1}^{N_{g}} \sum_{j=1}^{N_{s}} \frac{P\left(i,j\right)j^{2}}{i^{2}}}{N_{z}}$$

**Large Zone High Gray-Level Emphasis (LZHGE):**

$$LZHGE=\frac{\sum_{i=1}^{N_{g}} \sum_{j=1}^{N_{s}} P\left(i,j\right)i^{2}j^{2}}{N_{z}}$$

**Gray-Level Variance (GLV):**

$$GLV=\sum_{i=1}^{N_{g}} \sum_{j=1}^{N_{s}} p\left(i,j\right)\left(i-\sum_{i=1}^{N_{g}} \sum_{j=1}^{N_{s}} p\left(i,j\right)i\right)^{2}$$

**Zone-Size Variance (ZSV):**

$$ZSV=\sum_{i=1}^{N_{g}} \sum_{j=1}^{N_{s}} p\left(i,j\right)\left(j-\sum_{i=1}^{N_{g}} \sum_{j=1}^{N_{s}} p\left(i,j\right)j\right)^{2}$$

**Neighborhood gray-tone difference matrix (NGTDM):** NGTDMs were calculated for the entire 3D volume, which quantify the difference between a gray value and the average gray value of its neighbors. Let: $n_{i}$be the number of voxels in $X_{gl}$with gray level $i$, $p_{i}$be the gray level probability, $N_{g}$be the number of discreet gray levels, $N_{p}$be the total number of voxels in the ROI, $N_{g,p}$be the number of gray levels where $p_{i}\neq0$, $N_{v,p}$be the total number of voxels in $X_{gl}$, and $A_{i}$be the average gray level of the 26-connected neighbors around a center voxel with gray level $i$.

$$s_{i}=\left\{\begin{matrix} \sum_{i\in\left\{N_{i}\right\}} \left|i-\overline{A_{i}}\right|\text{ }if\text{ }n_{i}\neq0, \\ 0\text{ }otherwise \end{matrix}\right.$$

$$p_{i}=n_{i}/N_{v}$$

**Coarseness:**

$$Coarseness=\frac{1}{\sum_{i=1}^{N_{g}} p_{i}s_{i}}$$

**Contrast:**

$$contrast=\left(\frac{1}{N_{g,p}\left(N_{g,p}-1\right)}\sum_{i=1}^{N_{g}} \sum_{j=1}^{N_{g}} p_{i}p_{j}\left(i-j\right)^{2}\right)\left(\frac{1}{N_{v,p}}\sum_{i=1}^{N_{g}} s_{i}\right),where\text{ }p_{i}\neq0,p_{j}\neq0$$

**Busyness:**

$$Busyness=\frac{\sum_{i=1}^{N_{g}} p_{i}s_{i}}{\sum_{i=1}^{N_{g}} \sum_{j=1}^{N_{g}} \left|ip_{i}-jp_{j}\right|},where\text{ }p_{i}\neq0,p_{j}\neq0$$

**Complexity:**

$$Complexity=\frac{1}{N_{v,p}}\sum_{i=1}^{N_{g}} \sum_{j=1}^{N_{g}} \left|i-j\right|\frac{p_{i}s_{i}+p_{j}s_{j}}{p_{i}+p_{j}},where\text{ }p_{i}\neq0,p_{j}\neq0$$

**Strength:**

$$Strength=\frac{\sum_{i=1}^{N_{g}} \sum_{j=1}^{N_{g}} \left(p_{i}+p_{j}\right)\left(i-j\right)^{2}}{\sum_{i=1}^{N_{g}} s_{i}},where\text{ }p_{i}\neq0,p_{j}\neq0$$

**Appendix A2. The description of random forest method**

Random Forests (RFs) are designed to further improve the accuracy of models by building multiple trees i.e. a Forest. To make a prediction for a new observation, each tree in the forest gives a classification (a vote) on the outcome and the forest choose the classification having the majority votes (over all trees in the forest). To build a Random Forest, each constituent tree is forced to split on only a random subset of the available independent variables from a bootstrapped sample of the data. For example, the training data for each tree is selected randomly with replacement-since each tree uses different independent variables and different training data, we generate a Forest of different trees. The following figure provides an overview of the basic principle behind Random Forests.


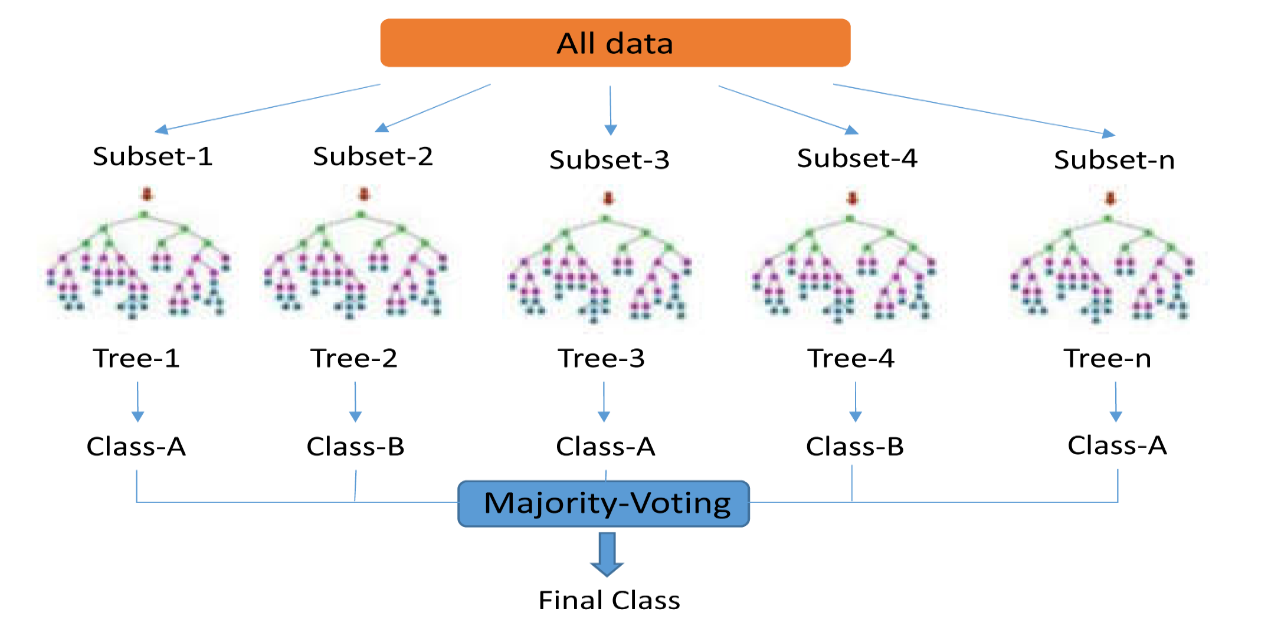


RF is a modification of bagging that builds and averages many trees to obtain an approximately unbiased models to reduce the variance. RF also has the ability to avoid the high variance or bias in prediction and is very simple to be implemented efficiently and accurately. Precisely, the RF will grow *B* trees to the bootstrapped data and repeating some statistical procedure to finally return the ensemble of the grown trees. When a new observation *x* is coming, let the
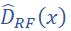
be the class prediction of the b-th tree, the

$$\mathop{D}\limits^{\wedge} _{RF}(\mathrm{x})=\mathrm{majority}\text{ }\mathrm{vote}\{\mathop{D^{b}}\limits^{\wedge} _{RF}(\mathrm{x})\}_{b=1}^{B}$$

**Appendix A3. Table 1. MRI examinations of RTLI-positive and RTLI-negative patients in models 1, 2 and 3**

| Prediction model | MRI examinations of RTLI-positive patients | MRI examinations of RTLI-negative patients | Total |
| --- | --- | --- | --- |
| Model 1 | 200 | 126 | 326 |
| Model 2 | 181 | 126 | 307 |
| Model 3 | 147 | 126 | 273 |

| **Appendix A4. Average AUC of selected radiomic features for three models** | | | | | | | | | |
| --- | --- | --- | --- | --- | --- | --- | --- | --- | --- |
| **Model 1** | | | | | | | | | |
|  | | | | Radios | Scale | Quantization | Gray levels | Texture type | **AUC** |
| **Medial temporal lobe** | CET1-w |  | 1 | | pixelW | Lloyd | 32 | NGTDM_Complexity | 0.7088 |
|  |  |  | 1 | | pixelW | Lloyd | 16 | NGTDM_Complexity | 0.6973 |
|  |  |  | 3/2 | | pixelW | Lloyd | 32 | NGTDM_Complexity | 0.6817 |
|  |  |  | 1 | | 1 | Lloyd | 64 | GLSZM_LGZE | 0.6754 |
|  |  |  | 2 | | 5 | Lloyd | 64 | GLSZM_SZLGE | 0.6727 |
|  |  |  | 3/2 | | 3 | Equal | 8 | Global_Kurtosis | 0.6708 |
|  |  |  | 3/2 | | 3 | Lloyd | 64 | Global_Kurtosis | 0.6704 |
|  |  |  | 3/2 | | 3 | Lloyd | 32 | Global_Kurtosis | 0.6700 |
|  |  |  | 3/2 | | 3 | Lloyd | 8 | Global_Kurtosis | 0.6699 |
|  |  |  | 3/2 | | 3 | Equal | 32 | Global_Kurtosis | 0.6698 |
|  |  |  | 3/2 | | 3 | Equal | 16 | Global_Kurtosis | 0.6696 |
|  |  |  | 2 | | 3 | Lloyd | 8 | GLSZM_LZE | 0.6620 |
|  |  |  | 3/2 | | 2 | Equal | 16 | GLSZM_GLV | 0.6549 |
|  |  |  | 1/2 | | 1 | Lloyd | 32 | GLSZM_LGZE | 0.6528 |
|  |  |  | 1/2 | | 4 | Lloyd | 16 | GLSZM_HGZE | 0.6517 |
|  |  |  | 3/2 | | pixelW | Lloyd | 16 | NGTDM_Complexity | 0.6501 |
|  |  |  | 1 | | 3 | Equal | 8 | GLCM_Variance | 0.6444 |
|  |  |  | 2/3 | | 3 | Equal | 16 | GLSZM_ZSV | 0.6400 |
|  |  |  | 1 | | 1 | Lloyd | 64 | GLSZM_SZLGE | 0.6386 |
|  |  |  | 2 | | pixelW | Lloyd | 16 | NGTDM_Complexity | 0.6318 |
|  | T2-w |  | 3/2 | | 1 | Lloyd | 64 | GLSZM_LZHGE | 0.7031 |
|  |  |  | 3/2 | | 2 | Lloyd | 64 | GLSZM_ZSV | 0.6959 |
|  |  |  | 1/2 | | 4 | Lloyd | 8 | GLRLM_SRLGE | 0.6900 |
|  |  |  | 1 | | 5 | Lloyd | 64 | GLSZM_LZHGE | 0.6829 |
|  |  |  | 3/2 | | 4 | Equal | 32 | GLSZM_SZE | 0.6731 |
|  |  |  | 3/2 | | 2 | Lloyd | 32 | GLCM_Energy | 0.6704 |
|  |  |  | 1/2 | | pixelW | Lloyd | 32 | GLCM_Sum Average | 0.6667 |
|  |  |  | 1 | | 4 | Lloyd | 32 | GLSZM_LZHGE | 0.6607 |
|  |  |  | 1 | | 4 | Lloyd | 64 | GLSZM_LZHGE | 0.6577 |
|  |  |  | 1 | | 2 | Equal | 32 | GLRLM_RLV | 0.6576 |
|  |  |  | 2 | | 2 | Lloyd | 16 | NGTDM_Coarseness | 0.6566 |
|  |  |  | 2/3 | | pixelW | Lloyd | 64 | GLRLM_GLV | 0.6565 |
|  |  |  | 2/3 | | 2 | Equal | 32 | GLRLM_RLV | 0.6551 |
|  |  |  | 1/2 | | 4 | Lloyd | 8 | GLRLM_LGRE | 0.6529 |
|  |  |  | 1 | | 4 | Equal | 32 | GLCM_Sum Average | 0.6527 |
|  |  |  | 2/3 | | 2 | Equal | 64 | Global_Skewness | 0.6526 |
|  |  |  | 2/3 | | 2 | Equal | 8 | Global_Skewness | 0.6510 |
|  |  |  | 2/3 | | 4 | Lloyd | 64 | GLCM_Sum Average | 0.6505 |
|  |  |  | 3/2 | | 5 | Lloyd | 8 | GLSZM_LZHGE | 0.6503 |
|  |  |  | 2/3 | | 2 | Lloyd | 8 | NGTDM_Strength | 0.6490 |
|  | CET1-w + T2-w | **CET1-w** | 1 | | pixelW | Lloyd | 32 | NGTDM_Complexity | 0.7075 |
|  |  | **T2-w** | 3/2 | | 1 | Lloyd | 64 | GLSZM_LZHGE | 0.7027 |
|  |  | **T2-w** | 1 | | 5 | Lloyd | 64 | GLSZM_LZHGE | 0.6821 |
|  |  | **T2-w** | 3/2 | | 4 | Equal | 32 | GLSZM_SZE | 0.6731 |
|  |  | **T2-w** | 3/2 | | 2 | Lloyd | 32 | GLCM_Energy | 0.6717 |
|  |  | **CET1-w** | 3/2 | | 3 | Lloyd | 8 | Global_Kurtosis | 0.6693 |
|  |  | **T2-w** | 2/3 | | 5 | Lloyd | 16 | GLRLM_LRHGE | 0.6685 |
|  |  | **T2-w** | 1 | | 4 | Lloyd | 32 | GLSZM_LZHGE | 0.6620 |
|  |  | **T2-w** | 1 | | 2 | Equal | 32 | GLRLM_RLV | 0.6593 |
|  |  | **T2-w** | 2/3 | | pixelW | Lloyd | 64 | GLRLM_GLV | 0.6567 |
|  |  | **T2-w** | 1 | | 4 | Lloyd | 64 | GLSZM_LZHGE | 0.6565 |
|  |  | **T2-w** | 2/3 | | 2 | Equal | 64 | Global_Skewness | 0.6518 |
|  |  | **T2-w** | 2/3 | | 2 | Equal | 8 | Global_Skewness | 0.6517 |
|  |  | **T2-w** | 2/3 | | 2 | Equal | 16 | Global_Skewness | 0.6511 |
|  |  | **T2-w** | 2/3 | | 2 | Lloyd | 32 | Global_Skewness | 0.6509 |
|  |  | **T2-w** | 2/3 | | 2 | Lloyd | 8 | Global_Skewness | 0.6499 |
|  |  | **T2-w** | 2/3 | | 4 | Lloyd | 64 | GLCM_Sum Average | 0.6492 |
|  |  | **T2-w** | 2/3 | | pixelW | Equal | 32 | GLSZM_SZLGE | 0.6466 |
|  |  | **T2-w** | 2 | | 2 | Equal | 16 | GLSZM_ZSV | 0.6447 |
|  |  | **T2-w** | 2 | | 2 | Lloyd | 16 | GLCM_Homogeneity | 0.6442 |
| **Gray matter** | **CET1-w** |  | 1 | | 5 | Lloyd | 16 | NGTDM_Coarseness | 0.6719 |
|  |  |  | 2 | | pixelW | Lloyd | 16 | GLSZM_SZHGE | 0.6579 |
|  |  |  | 3/2 | | 1 | Equal | 16 | GLSZM_ZSN | 0.6518 |
|  |  |  | 2/3 | | 5 | Lloyd | 16 | GLSZM_GLN | 0.6458 |
|  |  |  | 2/3 | | 1 | Lloyd | 16 | GLSZM_HGZE | 0.6312 |
|  |  |  | 2/3 | | 2 | Lloyd | 64 | GLSZM_GLV | 0.6274 |
|  |  |  | 2 | | 4 | Lloyd | 32 | GLRLM_RLV | 0.6186 |
|  |  |  | 1 | | pixelW | Equal | 8 | GLSZM_GLN | 0.6168 |
|  |  |  | 1/2 | | 2 | Lloyd | 32 | GLSZM_LGZE | 0.6162 |
|  |  |  | 3/2 | | 5 | Lloyd | 16 | NGTDM_Strength | 0.6155 |
|  |  |  | 2 | | 5 | Lloyd | 8 | NGTDM_Strength | 0.6145 |
|  |  |  | 2 | | 3 | Equal | 8 | GLSZM_GLN | 0.6131 |
|  |  |  | 1 | | pixelW | Equal | 32 | GLRLM_RLV | 0.6131 |
|  |  |  | 3/2 | | 4 | Equal | 8 | GLSZM_GLN | 0.6117 |
|  |  |  | 2 | | 3 | Lloyd | 16 | GLRLM_RLV | 0.6106 |
|  |  |  | 2 | | 3 | Lloyd | 16 | GLSZM_HGZE | 0.6100 |
|  |  |  | 1/2 | | 5 | Lloyd | 16 | GLRLM_LGRE | 0.6079 |
|  |  |  | 3/2 | | 2 | Equal | 32 | NGTDM_Coarseness | 0.6078 |
|  |  |  | 1/2 | | 3 | Lloyd | 8 | GLRLM_LRHGE | 0.6074 |
|  |  |  | 1 | | 4 | Equal | 8 | GLRLM_RLN | 0.6074 |
|  | **T2-w** |  | 2/3 | | 3 | Equal | 8 | GLSZM_GLV | 0.7207 |
|  |  |  | 1 | | 5 | Equal | 64 | NGTDM_Strength | 0.7203 |
|  |  |  | 3/2 | | 2 | Lloyd | 8 | GLSZM_GLV | 0.6972 |
|  |  |  | 1 | | 5 | Lloyd | 64 | NGTDM_Coarseness | 0.6931 |
|  |  |  | 2/3 | | 1 | Lloyd | 16 | GLRLM_LRHGE | 0.6796 |
|  |  |  | 1 | | 2 | Lloyd | 32 | GLRLM_RLV | 0.6709 |
|  |  |  | 2/3 | | 2 | Lloyd | 8 | GLSZM_GLV | 0.6700 |
|  |  |  | 1/2 | | pixelW | Lloyd | 16 | GLRLM_GLV | 0.6614 |
|  |  |  | 2/3 | | 2 | Equal | 16 | NGTDM_Strength | 0.6576 |
|  |  |  | 1 | | 2 | Equal | 8 | GLRLM_GLV | 0.6544 |
|  |  |  | 2/3 | | 2 | Equal | 8 | NGTDM_Strength | 0.6495 |
|  |  |  | 1/2 | | 2 | Equal | 8 | GLSZM_GLV | 0.6494 |
|  |  |  | 2 | | 2 | Lloyd | 16 | GLSZM_GLV | 0.6436 |
|  |  |  | 1 | | 4 | Equal | 32 | NGTDM_Strength | 0.6422 |
|  |  |  | 2/3 | | 1 | Equal | 8 | NGTDM_Strength | 0.6415 |
|  |  |  | 1 | | 2 | Equal | 16 | GLSZM_GLV | 0.6411 |
|  |  |  | 1 | | 4 | Equal | 16 | GLRLM_GLV | 0.6395 |
|  |  |  | 1 | | 3 | Lloyd | 32 | GLRLM_GLV | 0.6369 |
|  |  |  | 2/3 | | pixelW | Lloyd | 16 | GLSZM_GLV | 0.6356 |
|  |  |  | 3/2 | | 1 | Equal | 16 | GLRLM_GLV | 0.6348 |
|  | **CET1-w + T2-w** | **T2-w** | 2/3 | | 3 | Equal | 8 | GLSZM_GLV | 0.7214 |
|  |  | **T2-w** | 1 | | 5 | Lloyd | 16 | NGTDM_Coarseness | 0.7094 |
|  |  | **T2-w** | 3/2 | | 2 | Lloyd | 8 | GLSZM_GLV | 0.6972 |
|  |  | **T2-w** | 1 | | 5 | Lloyd | 64 | NGTDM_Coarseness | 0.6925 |
|  |  | **T2-w** | 1 | | 5 | Lloyd | 32 | NGTDM_Coarseness | 0.6886 |
|  |  | **T2-w** | 1 | | 2 | Lloyd | 32 | GLRLM_RLV | 0.6720 |
|  |  | **T2-w** | 2/3 | | 2 | Lloyd | 8 | GLSZM_GLV | 0.6700 |
|  |  | **T2-w** | 1 | | 3 | Lloyd | 64 | GLSZM_LZHGE | 0.6567 |
|  |  | **T2-w** | 1/2 | | 2 | Equal | 8 | GLSZM_GLV | 0.6489 |
|  |  | **T2-w** | 1 | | 3 | Equal | 32 | GLCM_Variance | 0.6453 |
|  |  | **T2-w** | 2 | | 3 | Lloyd | 32 | GLRLM_GLV | 0.6441 |
|  |  | **T2-w** | 2/3 | | 5 | Lloyd | 16 | GLCM_Auto Correlation | 0.6428 |
|  |  | **T2-w** | 1 | | 2 | Equal | 16 | GLSZM_GLV | 0.6410 |
|  |  | **T2-w** | 1 | | 4 | Equal | 16 | GLRLM_GLV | 0.6388 |
|  |  | **T2-w** | 2 | | 2 | Equal | 8 | NGTDM_Strength | 0.6337 |
|  |  | **T2-w** | 1 | | 5 | Equal | 16 | NGTDM_Strength | 0.6301 |
|  |  | **T2-w** | 1 | | 2 | Lloyd | 64 | GLSZM_GLV | 0.6301 |
|  |  | **T2-w** | 1 | | pixelW | Equal | 32 | GLSZM_GLV | 0.6296 |
|  |  | **T2-w** | 2 | | 2 | Lloyd | 32 | GLRLM_RLV | 0.6295 |
|  |  | **T2-w** | 3/2 | | 2 | Lloyd | 64 | GLRLM_RLV | 0.6287 |
| **white matter** | **CET1-w** |  | 1/2 | | pixelW | Equal | 64 | NGTDM_Busyness | 0.6591 |
|  |  |  | 1/2 | | 5 | Lloyd | 8 | GLRLM_LRLGE | 0.6560 |
|  |  |  | 3/2 | | 1 | Lloyd | 32 | GLSZM_LZLGE | 0.6454 |
|  |  |  | 2 | | 2 | Equal | 16 | GLRLM_RLV | 0.6392 |
|  |  |  | 2 | | pixelW | Lloyd | 16 | GLSZM_HGZE | 0.6316 |
|  |  |  | 1/2 | | 3 | Lloyd | 16 | GLSZM_LZE | 0.6304 |
|  |  |  | 2 | | 4 | Equal | 64 | GLSZM_SZE | 0.6294 |
|  |  |  | 3/2 | | 1 | Lloyd | 64 | GLSZM_LZLGE | 0.6271 |
|  |  |  | 1 | | 2 | Lloyd | 8 | GLRLM_RLV | 0.6246 |
|  |  |  | 1 | | pixelW | Equal | 64 | GLSZM_LZHGE | 0.6231 |
|  |  |  | 2 | | 3 | Equal | 8 | Global_Kurtosis | 0.6226 |
|  |  |  | 3/2 | | 3 | Equal | 8 | GLCM_Correlation | 0.6226 |
|  |  |  | 2 | | 3 | Lloyd | 8 | Global_Kurtosis | 0.6221 |
|  |  |  | 1 | | 2 | Lloyd | 32 | GLRLM_RLV | 0.6217 |
|  |  |  | 3/2 | | pixelW | Lloyd | 16 | GLSZM_LZLGE | 0.6212 |
|  |  |  | 2 | | 5 | Equal | 8 | GLRLM_RLV | 0.6210 |
|  |  |  | 1/2 | | 5 | Lloyd | 8 | GLRLM_LRE | 0.6187 |
|  |  |  | 1/2 | | 2 | Equal | 16 | GLRLM_LRLGE | 0.6166 |
|  |  |  | 1/2 | | 3 | Lloyd | 64 | GLSZM_LZE | 0.6160 |
|  |  |  | 2/3 | | 3 | Lloyd | 64 | GLSZM_LZLGE | 0.6157 |
|  | **T2-w** |  | 2 | | 2 | Lloyd | 32 | GLRLM_RLV | 0.6926 |
|  |  |  | 2 | | 3 | Equal | 8 | GLRLM_SRLGE | 0.6874 |
|  |  |  | 3/2 | | 3 | Equal | 32 | GLRLM_SRLGE | 0.6781 |
|  |  |  | 2/3 | | 1 | Equal | 16 | GLSZM_SZLGE | 0.6703 |
|  |  |  | 1 | | pixelW | Lloyd | 16 | GLRLM_LGRE | 0.6673 |
|  |  |  | 3/2 | | 4 | Equal | 8 | GLCM_Energy | 0.6666 |
|  |  |  | 2/3 | | 1 | Lloyd | 32 | GLRLM_LRLGE | 0.6664 |
|  |  |  | 2 | | 3 | Equal | 8 | GLCM_Energy | 0.6661 |
|  |  |  | 1/2 | | 1 | Lloyd | 32 | GLCM_Energy | 0.6630 |
|  |  |  | 1/2 | | 3 | Equal | 16 | GLRLM_SRLGE | 0.6590 |
|  |  |  | 2 | | 4 | Lloyd | 32 | GLSZM_LGZE | 0.6563 |
|  |  |  | 2/3 | | 1 | Equal | 16 | GLSZM_SZE | 0.6559 |
|  |  |  | 2 | | 2 | Equal | 16 | GLRLM_RLV | 0.6532 |
|  |  |  | 1 | | pixelW | Lloyd | 64 | GLRLM_LGRE | 0.6407 |
|  |  |  | 3/2 | | 2 | Lloyd | 32 | NGTDM_Strength | 0.6396 |
|  |  |  | 3/2 | | 5 | Lloyd | 32 | GLRLM_LRLGE | 0.6368 |
|  |  |  | 1/2 | | pixelW | Equal | 16 | GLRLM_SRLGE | 0.6331 |
|  |  |  | 1/2 | | 3 | Equal | 64 | GLRLM_SRLGE | 0.6317 |
|  |  |  | 2/3 | | pixelW | Equal | 16 | GLSZM_SZLGE | 0.6314 |
|  |  |  | 2/3 | | 1 | Equal | 8 | GLSZM_SZE | 0.6311 |
|  | **CET1-w +T2-w** | **T2-w** | 3/2 | | 3 | Equal | 8 | GLRLM_SRLGE | 0.7512 |
|  |  | **T2-w** | 2 | | 2 | Lloyd | 32 | GLRLM_RLV | 0.6927 |
|  |  | **T2-w** | 3/2 | | 3 | Equal | 16 | GLRLM_SRLGE | 0.6897 |
|  |  | **T2-w** | 2 | | 3 | Equal | 8 | GLRLM_SRLGE | 0.6876 |
|  |  | **T2-w** | 1/2 | | 1 | Equal | 8 | GLSZM_SZE | 0.6801 |
|  |  | **T2-w** | 3/2 | | 3 | Equal | 32 | GLRLM_SRLGE | 0.6782 |
|  |  | **T2-w** | 2/3 | | 3 | Equal | 64 | GLCM_Energy | 0.6723 |
|  |  | **T2-w** | 2/3 | | 1 | Equal | 16 | GLSZM_SZLGE | 0.6702 |
|  |  | **T1** | 2/3 | | pixelW | Lloyd | 8 | GLRLM_LRLGE | 0.6690 |
|  |  | **T2-w** | 2 | | 3 | Equal | 8 | GLCM_Energy | 0.6674 |
|  |  | **T2-w** | 1 | | pixelW | Lloyd | 16 | GLRLM_LGRE | 0.6661 |
|  |  | **T2-w** | 3/2 | | 4 | Equal | 8 | GLCM_Energy | 0.6652 |
|  |  | **CET1-w** | 1/2 | | pixelW | Equal | 64 | NGTDM_Busyness | 0.6598 |
|  |  | **T2-w** | 2/3 | | 1 | Equal | 16 | GLSZM_SZE | 0.6564 |
|  |  | **CET1-w** | 1/2 | | 1 | Equal | 32 | GLSZM_SZE | 0.6560 |
|  |  | **T2-w** | 3/2 | | 5 | Lloyd | 64 | GLRLM_SRLGE | 0.6550 |
|  |  | **CET1-w** | 3/2 | | 1 | Lloyd | 32 | GLSZM_LZLGE | 0.6467 |
|  |  | **T2-w** | 3/2 | | 3 | Equal | 64 | GLRLM_SRLGE | 0.6455 |
|  |  | **T2-w** | 3/2 | | 5 | Lloyd | 64 | GLRLM_LGRE | 0.6430 |
|  |  | **T2-w** | 3/2 | | pixelW | Lloyd | 8 | GLRLM_LGRE | 0.6418 |

| **Model 2** | | | | | | | | |
| --- | --- | --- | --- | --- | --- | --- | --- | --- |
|  | | | Radios | Scale | Quantization | Gray levels | Texture type | **AUC** |
| **Medial temporal lobe** | **CET1-w** |  | 1 | 2 | Equal | 32 | GLSZM_SZE | 0.6682 |
|  |  |  | 2/3 | 5 | Equal | 8 | GLRLM_GLN | 0.6625 |
|  |  |  | 1/2 | 5 | Lloyd | 32 | GLSZM_SZE | 0.6407 |
|  |  |  | 2 | 4 | Lloyd | 64 | GLSZM_SZLGE | 0.6400 |
|  |  |  | 1 | 3 | Lloyd | 32 | GLSZM_LGZE | 0.6385 |
|  |  |  | 1 | 2 | Lloyd | 32 | GLSZM_SZLGE | 0.6371 |
|  |  |  | 1/2 | 4 | Equal | 8 | GLSZM_GLN | 0.6370 |
|  |  |  | 1 | 4 | Lloyd | 64 | GLSZM_LGZE | 0.6337 |
|  |  |  | 2/3 | 1 | Equal | 32 | NGTDM_Busyness | 0.6336 |
|  |  |  | 1 | 1 | Lloyd | 32 | GLSZM_LGZE | 0.6330 |
|  |  |  | 3/2 | 3 | Equal | 8 | GLRLM_RLV | 0.6319 |
|  |  |  | 2/3 | pixelW | Equal | 16 | GLCM_Sum Average | 0.6307 |
|  |  |  | 2 | 2 | Lloyd | 16 | GLSZM_SZLGE | 0.6302 |
|  |  |  | 1 | 5 | Lloyd | 8 | GLSZM_LZE | 0.6272 |
|  |  |  | 3/2 | 2 | Lloyd | 16 | GLRLM_SRLGE | 0.6267 |
|  |  |  | 2/3 | pixelW | Lloyd | 8 | GLSZM_GLN | 0.6262 |
|  |  |  | 2 | 4 | Lloyd | 32 | GLSZM_SZLGE | 0.6262 |
|  |  |  | 3/2 | 4 | Lloyd | 8 | GLSZM_SZLGE | 0.6256 |
|  |  |  | 3/2 | 2 | Lloyd | 8 | GLSZM_SZHGE | 0.6252 |
|  |  |  | 3/2 | 4 | Lloyd | 8 | GLSZM_SZE | 0.6249 |
|  | **T2-w** |  | 2 | 2 | Equal | 16 | GLSZM_ZSV | 0.7372 |
|  |  |  | 2/3 | 4 | Lloyd | 16 | Global_Skewness | 0.7163 |
|  |  |  | 2/3 | 4 | Equal | 32 | Global_Skewness | 0.7162 |
|  |  |  | 2/3 | 4 | Equal | 64 | Global_Skewness | 0.7153 |
|  |  |  | 2/3 | 4 | Lloyd | 32 | Global_Skewness | 0.7149 |
|  |  |  | 2/3 | 4 | Equal | 8 | Global_Skewness | 0.7146 |
|  |  |  | 2/3 | 4 | Equal | 16 | Global_Skewness | 0.7144 |
|  |  |  | 2/3 | 4 | Lloyd | 8 | Global_Skewness | 0.7143 |
|  |  |  | 2/3 | 4 | Lloyd | 64 | Global_Skewness | 0.7130 |
|  |  |  | 2/3 | 1 | Lloyd | 32 | GLSZM_SZHGE | 0.7129 |
|  |  |  | 1 | 1 | Lloyd | 8 | GLRLM_LRHGE | 0.7055 |
|  |  |  | 3/2 | 4 | Lloyd | 8 | GLRLM_LRHGE | 0.6970 |
|  |  |  | 2/3 | 5 | Equal | 8 | GLCM_Auto Correlation | 0.6943 |
|  |  |  | 1/2 | 4 | Equal | 8 | Global_Skewness | 0.6899 |
|  |  |  | 1/2 | 4 | Lloyd | 32 | Global_Skewness | 0.6897 |
|  |  |  | 1/2 | 4 | Equal | 64 | Global_Skewness | 0.6895 |
|  |  |  | 1/2 | 4 | Lloyd | 64 | Global_Skewness | 0.6891 |
|  |  |  | 1/2 | 4 | Equal | 32 | Global_Skewness | 0.6890 |
|  |  |  | 3/2 | 4 | Lloyd | 64 | GLSZM_LZHGE | 0.6880 |
|  |  |  | 3/2 | 4 | Equal | 8 | GLSZM_ZSV | 0.6872 |
|  | **CET1-w+T2-w** | T2-w | 2 | 2 | Equal | 16 | GLSZM_ZSV | 0.7382 |
|  |  | T2-w | 2/3 | 4 | Equal | 32 | Global_Skewness | 0.7158 |
|  |  | T2-w | 2/3 | 4 | Equal | 8 | Global_Skewness | 0.7153 |
|  |  | T2-w | 2/3 | 4 | Lloyd | 8 | Global_Skewness | 0.7151 |
|  |  | T2-w | 2/3 | 4 | Lloyd | 16 | Global_Skewness | 0.7145 |
|  |  | T2-w | 2/3 | 4 | Equal | 64 | Global_Skewness | 0.7143 |
|  |  | T2-w | 2/3 | 4 | Lloyd | 64 | Global_Skewness | 0.7139 |
|  |  | T2-w | 2/3 | 4 | Lloyd | 32 | Global_Skewness | 0.7139 |
|  |  | T2-w | 2/3 | 4 | Equal | 16 | Global_Skewness | 0.7139 |
|  |  | T2-w | 1 | 1 | Lloyd | 8 | GLRLM_LRHGE | 0.7047 |
|  |  | T2-w | 3/2 | 4 | Lloyd | 8 | GLRLM_LRHGE | 0.6975 |
|  |  | T2-w | 1/2 | 4 | Equal | 64 | Global_Skewness | 0.6907 |
|  |  | T2-w | 1/2 | 4 | Equal | 32 | Global_Skewness | 0.6899 |
|  |  | T2-w | 1/2 | 4 | Equal | 16 | Global_Skewness | 0.6893 |
|  |  | T2-w | 1/2 | 4 | Lloyd | 8 | Global_Skewness | 0.6893 |
|  |  | T2-w | 1/2 | 4 | Lloyd | 64 | Global_Skewness | 0.6888 |
|  |  | T2-w | 2 | 4 | Lloyd | 64 | GLSZM_LZHGE | 0.6878 |
|  |  | T2-w | 3/2 | 4 | Equal | 8 | GLSZM_ZSV | 0.6875 |
|  |  | T2-w | 2 | 5 | Equal | 64 | GLCM_Correlation | 0.6874 |
|  |  | T2-w | 1/2 | 1 | Lloyd | 8 | GLRLM_HGRE | 0.6862 |
| **Gray matter** | **CET1-w** |  | 2/3 | pixelW | Lloyd | 64 | GLRLM_LRHGE | 0.6729 |
|  |  |  | 3/2 | 5 | Lloyd | 64 | Global_Kurtosis | 0.6657 |
|  |  |  | 1/2 | pixelW | Equal | 16 | GLSZM_GLN | 0.6647 |
|  |  |  | 2/3 | pixelW | Equal | 64 | GLSZM_LZHGE | 0.6421 |
|  |  |  | 2/3 | 1 | Lloyd | 8 | GLSZM_LZHGE | 0.6385 |
|  |  |  | 2/3 | 5 | Equal | 32 | GLSZM_ZSN | 0.6339 |
|  |  |  | 3/2 | 1 | Lloyd | 8 | GLSZM_LZHGE | 0.6338 |
|  |  |  | 1 | 1 | Lloyd | 16 | GLSZM_LZHGE | 0.6309 |
|  |  |  | 2/3 | 2 | Lloyd | 32 | GLSZM_LZHGE | 0.6294 |
|  |  |  | 3/2 | pixelW | Equal | 8 | GLSZM_GLN | 0.6279 |
|  |  |  | 3/2 | 5 | Equal | 8 | GLSZM_GLN | 0.6258 |
|  |  |  | 1 | 1 | Lloyd | 32 | GLSZM_LZHGE | 0.6251 |
|  |  |  | 2/3 | 4 | Equal | 32 | GLSZM_LZHGE | 0.6241 |
|  |  |  | 2/3 | 5 | Equal | 8 | GLRLM_RLV | 0.6235 |
|  |  |  | 2/3 | 1 | Lloyd | 8 | Global_Kurtosis | 0.6225 |
|  |  |  | 2/3 | 1 | Equal | 16 | Global_Kurtosis | 0.6215 |
|  |  |  | 2/3 | 3 | Equal | 8 | GLSZM_ZSN | 0.6206 |
|  |  |  | 1 | 4 | Lloyd | 8 | GLRLM_GLV | 0.6193 |
|  |  |  | 1 | 2 | Lloyd | 32 | GLSZM_LGZE | 0.6179 |
|  |  |  | 1 | 1 | Lloyd | 8 | NGTDM_Busyness | 0.6149 |
|  | **T2-w** |  | 2 | pixelW | Equal | 32 | GLRLM_GLV | 0.6674 |
|  |  |  | 3/2 | pixelW | Lloyd | 64 | GLRLM_GLV | 0.6609 |
|  |  |  | 3/2 | 2 | Equal | 32 | GLSZM_GLV | 0.6568 |
|  |  |  | 1/2 | 4 | Lloyd | 64 | GLRLM_GLV | 0.6537 |
|  |  |  | 1/2 | 2 | Equal | 16 | GLSZM_GLV | 0.6459 |
|  |  |  | 1 | 3 | Lloyd | 64 | GLSZM_LZHGE | 0.6440 |
|  |  |  | 2 | 2 | Equal | 8 | GLSZM_GLV | 0.6427 |
|  |  |  | 2 | 2 | Lloyd | 16 | GLRLM_GLV | 0.6421 |
|  |  |  | 1 | 5 | Lloyd | 16 | GLRLM_RLV | 0.6411 |
|  |  |  | 1 | pixelW | Equal | 32 | GLSZM_GLV | 0.6352 |
|  |  |  | 1/2 | 2 | Equal | 64 | GLRLM_RLV | 0.6335 |
|  |  |  | 1 | 4 | Lloyd | 32 | GLRLM_RLV | 0.6316 |
|  |  |  | 2/3 | 5 | Lloyd | 8 | GLRLM_RLV | 0.6314 |
|  |  |  | 1 | pixelW | Equal | 16 | GLSZM_ZSN | 0.6307 |
|  |  |  | 2 | 2 | Equal | 64 | GLRLM_RLV | 0.6282 |
|  |  |  | 2/3 | 2 | Equal | 16 | GLSZM_GLV | 0.6277 |
|  |  |  | 1/2 | pixelW | Lloyd | 16 | GLRLM_GLV | 0.6258 |
|  |  |  | 3/2 | 1 | Lloyd | 16 | GLSZM_GLV | 0.6238 |
|  |  |  | 2/3 | 1 | Equal | 16 | GLRLM_GLV | 0.6230 |
|  |  |  | 3/2 | 4 | Equal | 64 | GLSZM_GLV | 0.6229 |
|  | **CET1-w+T2-w** | T2-w | 2 | pixelW | Equal | 32 | GLRLM_GLV | 0.6658 |
|  |  | T2-w | 3/2 | pixelW | Lloyd | 64 | GLRLM_GLV | 0.6612 |
|  |  | T2-w | 3/2 | 2 | Equal | 32 | GLSZM_GLV | 0.6565 |
|  |  | T2-w | 1/2 | 2 | Equal | 16 | GLSZM_GLV | 0.6458 |
|  |  | CET1-w | 1/2 | 5 | Equal | 16 | GLSZM_ZSN | 0.6451 |
|  |  | T2-w | 1 | 3 | Lloyd | 64 | GLSZM_LZHGE | 0.6437 |
|  |  | T2-w | 1/2 | 2 | Equal | 32 | GLRLM_GLV | 0.6424 |
|  |  | CET1-w | 2/3 | 1 | Lloyd | 8 | GLSZM_LZHGE | 0.6391 |
|  |  | T2-w | 1/2 | 2 | Equal | 64 | GLRLM_RLV | 0.6344 |
|  |  | CET1-w | 1 | 1 | Lloyd | 16 | GLSZM_LZHGE | 0.6307 |
|  |  | CET1-w | 2/3 | 2 | Lloyd | 32 | GLSZM_LZHGE | 0.6303 |
|  |  | CET1-w | 3/2 | pixelW | Equal | 8 | GLSZM_GLN | 0.6302 |
|  |  | T2-w | 2 | 2 | Equal | 64 | GLRLM_RLV | 0.6300 |
|  |  | T2-w | 1 | pixelW | Equal | 16 | GLSZM_ZSN | 0.6297 |
|  |  | T2-w | 2/3 | 2 | Equal | 16 | GLSZM_GLV | 0.6259 |
|  |  | CET1-w | 1 | 1 | Lloyd | 32 | GLSZM_LZHGE | 0.6253 |
|  |  | CET1-w | 2/3 | 5 | Equal | 8 | GLRLM_RLV | 0.6228 |
|  |  | CET1-w | 2/3 | 1 | Lloyd | 8 | Global_Kurtosis | 0.6225 |
|  |  | CET1-w | 2/3 | 1 | Equal | 16 | Global_Kurtosis | 0.6218 |
|  |  | T2-w | 1 | 2 | Equal | 64 | GLRLM_GLV | 0.6216 |
| **White matter** | **CET1-w** |  | 2/3 | 2 | Equal | 8 | Global_Skewness | 0.6626 |
|  |  |  | 1 | pixelW | Lloyd | 16 | GLSZM_SZLGE | 0.6529 |
|  |  |  | 2 | 2 | Equal | 64 | GLSZM_ZSV | 0.6508 |
|  |  |  | 3/2 | 3 | Lloyd | 8 | Global_Skewness | 0.6501 |
|  |  |  | 3/2 | 3 | Equal | 8 | Global_Skewness | 0.6487 |
|  |  |  | 1 | 1 | Lloyd | 16 | GLRLM_LRLGE | 0.6425 |
|  |  |  | 1/2 | 2 | Equal | 16 | GLCM_Correlation | 0.6399 |
|  |  |  | 2/3 | 5 | Equal | 16 | GLSZM_LZLGE | 0.6381 |
|  |  |  | 2/3 | pixelW | Equal | 16 | NGTDM_Busyness | 0.6236 |
|  |  |  | 1 | 1 | Equal | 8 | Global_Variance | 0.6225 |
|  |  |  | 3/2 | 4 | Lloyd | 8 | GLCM_Correlation | 0.6224 |
|  |  |  | 1 | 1 | Equal | 32 | Global_Variance | 0.6223 |
|  |  |  | 1/2 | 5 | Equal | 64 | GLSZM_LZE | 0.6222 |
|  |  |  | 1 | 1 | Equal | 64 | Global_Variance | 0.6220 |
|  |  |  | 3/2 | 2 | Equal | 8 | Global_Skewness | 0.6212 |
|  |  |  | 3/2 | 1 | Equal | 16 | Global_Skewness | 0.6202 |
|  |  |  | 2 | 1 | Lloyd | 16 | Global_Kurtosis | 0.6200 |
|  |  |  | 2 | 1 | Equal | 8 | Global_Kurtosis | 0.6197 |
|  |  |  | 2 | 1 | Equal | 16 | Global_Kurtosis | 0.6196 |
|  |  |  | 2 | 1 | Lloyd | 64 | Global_Kurtosis | 0.6194 |
|  | **T2-w** |  | 2 | 1 | Lloyd | 64 | GLSZM_ZSV | 0.6834 |
|  |  |  | 2 | pixelW | Lloyd | 32 | GLSZM_ZSV | 0.6739 |
|  |  |  | 1 | 4 | Equal | 32 | GLSZM_SZE | 0.6657 |
|  |  |  | 1/2 | 1 | Lloyd | 16 | GLSZM_LGZE | 0.6649 |
|  |  |  | 1/2 | pixelW | Lloyd | 32 | GLRLM_LRLGE | 0.6610 |
|  |  |  | 1/2 | 2 | Equal | 32 | GLSZM_ZSV | 0.6578 |
|  |  |  | 2/3 | 2 | Equal | 32 | GLSZM_ZSV | 0.6503 |
|  |  |  | 3/2 | 2 | Equal | 8 | GLSZM_ZSV | 0.6457 |
|  |  |  | 3/2 | 2 | Lloyd | 8 | GLSZM_ZSV | 0.6432 |
|  |  |  | 1 | 2 | Lloyd | 16 | GLRLM_RLV | 0.6425 |
|  |  |  | 2/3 | pixelW | Equal | 64 | Global_Kurtosis | 0.6402 |
|  |  |  | 2/3 | pixelW | Equal | 8 | Global_Kurtosis | 0.6390 |
|  |  |  | 3/2 | 4 | Lloyd | 16 | GLCM_Correlation | 0.6382 |
|  |  |  | 1 | 4 | Lloyd | 64 | GLRLM_LGRE | 0.6356 |
|  |  |  | 1 | 2 | Lloyd | 8 | GLSZM_ZSV | 0.6349 |
|  |  |  | 2/3 | pixelW | Lloyd | 16 | GLRLM_LRLGE | 0.6346 |
|  |  |  | 2/3 | 3 | Equal | 64 | Global_Kurtosis | 0.6333 |
|  |  |  | 1/2 | pixelW | Equal | 32 | GLSZM_ZSV | 0.6322 |
|  |  |  | 3/2 | pixelW | Equal | 16 | GLSZM_ZSV | 0.6295 |
|  |  |  | 1/2 | 2 | Equal | 16 | GLSZM_SZLGE | 0.6275 |
|  | **CET1-w+T2-w** | T2-w | 2 | pixelW | Lloyd | 32 | GLSZM_ZSV | 0.6746 |
|  |  | T2-w | 3/2 | 1 | Lloyd | 16 | GLRLM_LGRE | 0.6690 |
|  |  | T2-w | 1/2 | 1 | Lloyd | 16 | GLSZM_LGZE | 0.6654 |
|  |  | T2-w | 1/2 | pixelW | Lloyd | 32 | GLRLM_LRLGE | 0.6615 |
|  |  | T2-w | 1/2 | 2 | Equal | 32 | GLSZM_ZSV | 0.6573 |
|  |  | CET1-w | 2/3 | 4 | Lloyd | 8 | GLSZM_GLN | 0.6521 |
|  |  | T2-w | 2/3 | 2 | Equal | 32 | GLSZM_ZSV | 0.6510 |
|  |  | T2-w | 3/2 | 2 | Lloyd | 8 | GLSZM_ZSV | 0.6402 |
|  |  | CET1-w | 1/2 | 2 | Equal | 16 | GLCM_Correlation | 0.6386 |
|  |  | T2-w | 1 | 2 | Lloyd | 8 | GLSZM_ZSV | 0.6343 |
|  |  | T2-w | 2/3 | pixelW | Lloyd | 16 | GLRLM_LRLGE | 0.6343 |
|  |  | T2-w | 1/2 | pixelW | Equal | 32 | GLSZM_ZSV | 0.6326 |
|  |  | T2-w | 2 | 1 | Lloyd | 32 | GLSZM_ZSV | 0.6259 |
|  |  | CET1-w | 2/3 | pixelW | Equal | 16 | NGTDM_Busyness | 0.6255 |
|  |  | T2-w | 2 | 1 | Lloyd | 16 | GLSZM_ZSV | 0.6237 |
|  |  | T2-w | 3/2 | 4 | Equal | 8 | Global_Variance | 0.6233 |
|  |  | CET1-w | 3/2 | 4 | Lloyd | 8 | GLCM_Correlation | 0.6227 |
|  |  | CET1-w | 1 | 1 | Equal | 8 | Global_Variance | 0.6218 |
|  |  | CET1-w | 2/3 | 3 | Lloyd | 8 | GLSZM_HGZE | 0.6205 |
|  |  | CET1-w | 2 | 1 | Equal | 32 | Global_Kurtosis | 0.6203 |

| **Model 3** | | | | | | | | | |
| --- | --- | --- | --- | --- | --- | --- | --- | --- | --- |
|  | | | Radios | Scale | Quantization | Gray levels | | Texture type | **AUC** |
| **Medial temporal lobe** | **CET1-w** |  | 1/2 | pixelW | Equal | 16 | NGTDM_Complexity | | 0.7048 |
|  |  |  | 1 | 3 | Equal | 8 | GLCM_Variance | | 0.7005 |
|  |  |  | 2 | 1 | Lloyd | 32 | GLSZM_SZLGE | | 0.6931 |
|  |  |  | 1 | 1 | Equal | 64 | GLRLM_GLN | | 0.6807 |
|  |  |  | 1/2 | 5 | Lloyd | 64 | GLCM_Energy | | 0.6804 |
|  |  |  | 3/2 | pixelW | Lloyd | 64 | GLSZM_LGZE | | 0.6796 |
|  |  |  | 1/2 | 1 | Equal | 32 | GLCM_Auto Correlation | | 0.6571 |
|  |  |  | 2/3 | 5 | Lloyd | 64 | GLCM_Energy | | 0.6552 |
|  |  |  | 3/2 | 4 | Equal | 16 | GLRLM_GLV | | 0.6549 |
|  |  |  | 1 | 4 | Lloyd | 16 | GLRLM_SRE | | 0.6537 |
|  |  |  | 3/2 | 1 | Equal | 32 | Global_Kurtosis | | 0.6526 |
|  |  |  | 3/2 | 1 | Equal | 64 | Global_Kurtosis | | 0.6520 |
|  |  |  | 3/2 | 1 | Lloyd | 64 | GLSZM_SZE | | 0.6514 |
|  |  |  | 3/2 | 1 | Lloyd | 32 | Global_Kurtosis | | 0.6512 |
|  |  |  | 2/3 | 1 | Equal | 8 | GLCM_Variance | | 0.6500 |
|  |  |  | 3/2 | 3 | Equal | 64 | Global_Kurtosis | | 0.6467 |
|  |  |  | 2/3 | 1 | Equal | 64 | GLRLM_GLN | | 0.6463 |
|  |  |  | 1/2 | 3 | Equal | 32 | GLRLM_HGRE | | 0.6458 |
|  |  |  | 1 | 4 | Equal | 32 | GLSZM_LZLGE | | 0.6452 |
|  |  |  | 2/3 | 5 | Equal | 16 | Global_Kurtosis | | 0.6435 |
|  | **T2-w** |  | 3/2 | 4 | Lloyd | 16 | Global_Kurtosis | | 0.7001 |
|  |  |  | 1/2 | pixelW | Equal | 32 | GLRLM_LRLGE | | 0.6741 |
|  |  |  | 2 | pixelW | Lloyd | 8 | GLRLM_HGRE | | 0.6664 |
|  |  |  | 1/2 | pixelW | Lloyd | 16 | GLSZM_SZLGE | | 0.6663 |
|  |  |  | 1/2 | 4 | Lloyd | 8 | GLRLM_LGRE | | 0.6645 |
|  |  |  | 1 | 3 | Equal | 32 | GLRLM_LRLGE | | 0.6591 |
|  |  |  | 2/3 | 1 | Lloyd | 32 | GLRLM_RLV | | 0.6577 |
|  |  |  | 3/2 | 4 | Equal | 64 | GLSZM_LGZE | | 0.6539 |
|  |  |  | 3/2 | 5 | Equal | 8 | GLSZM_SZE | | 0.6490 |
|  |  |  | 3/2 | 1 | Lloyd | 32 | GLRLM_HGRE | | 0.6490 |
|  |  |  | 1/2 | 1 | Equal | 16 | GLRLM_LRLGE | | 0.6464 |
|  |  |  | 1/2 | 1 | Lloyd | 8 | GLRLM_LGRE | | 0.6459 |
|  |  |  | 1/2 | 1 | Lloyd | 8 | GLRLM_SRLGE | | 0.6451 |
|  |  |  | 2/3 | pixelW | Lloyd | 16 | GLSZM_SZLGE | | 0.6417 |
|  |  |  | 1 | 1 | Equal | 32 | Global_Skewness | | 0.6409 |
|  |  |  | 1 | 1 | Equal | 8 | Global_Skewness | | 0.6407 |
|  |  |  | 1 | pixelW | Equal | 32 | Global_Skewness | | 0.6405 |
|  |  |  | 1 | pixelW | Equal | 64 | Global_Skewness | | 0.6401 |
|  |  |  | 1 | 1 | Equal | 16 | Global_Skewness | | 0.6399 |
|  |  |  | 1 | 1 | Equal | 64 | Global_Skewness | | 0.6399 |
|  | **CET1-w +T2-w** | T2-w | 1/2 | 3 | Lloyd | 8 | GLRLM_LGRE | | 0.7444 |
|  |  | CET1-w | 1/2 | pixelW | Equal | 16 | NGTDM_Complexity | | 0.7062 |
|  |  | CET1-w | 2 | 1 | Lloyd | 32 | GLSZM_SZLGE | | 0.6914 |
|  |  | T2-w | 2/3 | 1 | Equal | 16 | GLRLM_LGRE | | 0.6861 |
|  |  | T2-w | 1 | pixelW | Equal | 16 | GLCM_Sum Average | | 0.6844 |
|  |  | CET1-w | 2/3 | 4 | Equal | 8 | Global_Kurtosis | | 0.6692 |
|  |  | T2-w | 2 | 2 | Lloyd | 64 | GLSZM_SZHGE | | 0.6670 |
|  |  | T2-w | 1/2 | 4 | Lloyd | 8 | GLRLM_LGRE | | 0.6643 |
|  |  | T2-w | 2/3 | 1 | Lloyd | 32 | GLRLM_RLV | | 0.6585 |
|  |  | T2-w | 1/2 | pixelW | Lloyd | 32 | GLCM_Sum Average | | 0.6550 |
|  |  | T2-w | 3/2 | 4 | Equal | 64 | GLSZM_LGZE | | 0.6545 |
|  |  | CET1-w | 1/2 | 2 | Lloyd | 8 | GLCM_Energy | | 0.6536 |
|  |  | T2-w | 1 | 1 | Equal | 8 | GLRLM_GLV | | 0.6524 |
|  |  | CET1-w | 3/2 | 1 | Equal | 64 | Global_Kurtosis | | 0.6518 |
|  |  | CET1-w | 3/2 | 1 | Lloyd | 64 | GLSZM_SZE | | 0.6512 |
|  |  | CET1-w | 3/2 | 1 | Equal | 32 | Global_Kurtosis | | 0.6511 |
|  |  | T2-w | 3/2 | 5 | Equal | 8 | GLSZM_SZE | | 0.6504 |
|  |  | CET1-w | 2/3 | 1 | Equal | 8 | GLCM_Variance | | 0.6498 |
|  |  | CET1-w | 1 | pixelW | Lloyd | 8 | GLSZM_ZSN | | 0.6477 |
|  |  | T2-w | 1/2 | 1 | Lloyd | 8 | GLRLM_SRLGE | | 0.6473 |
| **Gray matter** | **CET1-w** |  | 3/2 | 4 | Lloyd | 16 | NGTDM_Coarseness | | 0.6974 |
|  |  |  | 1 | 2 | Lloyd | 32 | GLSZM_SZE | | 0.6858 |
|  |  |  | 1 | 2 | Equal | 64 | GLSZM_GLV | | 0.6748 |
|  |  |  | 1/2 | pixelW | Lloyd | 16 | GLRLM_LRHGE | | 0.6699 |
|  |  |  | 2 | 1 | Equal | 32 | GLRLM_RLV | | 0.6684 |
|  |  |  | 2 | 2 | Lloyd | 32 | GLRLM_RLV | | 0.6650 |
|  |  |  | 1 | 4 | Equal | 16 | GLRLM_RLN | | 0.6598 |
|  |  |  | 3/2 | 2 | Lloyd | 32 | GLSZM_ZSV | | 0.6568 |
|  |  |  | 1 | 2 | Equal | 16 | GLSZM_ZSV | | 0.6553 |
|  |  |  | 2 | 3 | Equal | 16 | GLSZM_ZSV | | 0.6543 |
|  |  |  | 1/2 | 5 | Equal | 64 | GLRLM_RLV | | 0.6506 |
|  |  |  | 3/2 | 3 | Equal | 32 | GLSZM_ZSV | | 0.6489 |
|  |  |  | 2/3 | 3 | Equal | 16 | GLSZM_GLV | | 0.6479 |
|  |  |  | 3/2 | 4 | Lloyd | 8 | GLSZM_GLV | | 0.6463 |
|  |  |  | 2/3 | 3 | Lloyd | 64 | GLSZM_GLV | | 0.6457 |
|  |  |  | 3/2 | 1 | Equal | 8 | GLSZM_ZSN | | 0.6445 |
|  |  |  | 2 | 4 | Equal | 64 | GLRLM_RLV | | 0.6424 |
|  |  |  | 1/2 | 1 | Equal | 32 | GLSZM_LZHGE | | 0.6407 |
|  |  |  | 2 | 2 | Equal | 8 | GLRLM_GLV | | 0.6390 |
|  |  |  | 3/2 | 3 | Lloyd | 64 | GLSZM_GLV | | 0.6385 |
|  |  |  |  |  |  |  |  |  |  |
|  | **T2-w** |  | 1 | 2 | Equal | 16 | GLSZM_GLV | | 0.7088 |
|  |  |  | 2/3 | 2 | Equal | 16 | GLSZM_GLV | | 0.7032 |
|  |  |  | 2/3 | 1 | Equal | 8 | GLSZM_GLN | | 0.6917 |
|  |  |  | 1/2 | 2 | Equal | 16 | GLSZM_GLV | | 0.6890 |
|  |  |  | 1 | 1 | Equal | 32 | GLRLM_GLV | | 0.6847 |
|  |  |  | 1/2 | 2 | Lloyd | 64 | GLRLM_RLV | | 0.6840 |
|  |  |  | 2/3 | 2 | Lloyd | 64 | GLRLM_GLV | | 0.6808 |
|  |  |  | 3/2 | pixelW | Lloyd | 32 | GLRLM_RLV | | 0.6792 |
|  |  |  | 3/2 | 2 | Equal | 32 | GLRLM_RLV | | 0.6789 |
|  |  |  | 1/2 | 2 | Equal | 8 | GLSZM_ZSV | | 0.6751 |
|  |  |  | 1 | 3 | Equal | 64 | GLSZM_LZHGE | | 0.6744 |
|  |  |  | 1/2 | 2 | Equal | 32 | GLRLM_RLV | | 0.6721 |
|  |  |  | 3/2 | 4 | Equal | 64 | GLRLM_RLV | | 0.6672 |
|  |  |  | 1/2 | 2 | Equal | 16 | GLRLM_RLV | | 0.6663 |
|  |  |  | 1/2 | 2 | Lloyd | 32 | GLRLM_RLV | | 0.6649 |
|  |  |  | 2/3 | 5 | Equal | 8 | GLSZM_ZSN | | 0.6643 |
|  |  |  | 3/2 | 3 | Equal | 8 | GLSZM_ZSN | | 0.6639 |
|  |  |  | 1/2 | 3 | Equal | 16 | GLRLM_RLV | | 0.6631 |
|  |  |  | 1 | 5 | Equal | 32 | GLSZM_GLV | | 0.6628 |
|  |  |  | 2/3 | 2 | Equal | 16 | GLRLM_RLV | | 0.6591 |
|  | **CET1-w+T2-w** | T2-w | 2 | 3 | Lloyd | 64 | GLRLM_GLV | | 0.7801 |
|  |  | T2-w | 2/3 | 2 | Equal | 16 | GLSZM_GLV | | 0.7040 |
|  |  | T2-w | 3/2 | 1 | Lloyd | 64 | GLRLM_GLV | | 0.6982 |
|  |  | T2-w | 2/3 | 1 | Equal | 8 | GLSZM_GLN | | 0.6910 |
|  |  | T2-w | 1/2 | 2 | Equal | 16 | GLSZM_GLV | | 0.6896 |
|  |  | T2-w | 3/2 | 2 | Lloyd | 64 | GLRLM_GLV | | 0.6836 |
|  |  | T2-w | 1/2 | 2 | Lloyd | 64 | GLRLM_RLV | | 0.6834 |
|  |  | T2-w | 2/3 | 2 | Lloyd | 64 | GLRLM_GLV | | 0.6809 |
|  |  | T2-w | 3/2 | 2 | Equal | 32 | GLRLM_RLV | | 0.6798 |
|  |  | T2-w | 3/2 | pixelW | Lloyd | 32 | GLRLM_RLV | | 0.6791 |
|  |  | CET1-w | 1 | 2 | Equal | 64 | GLSZM_GLV | | 0.6757 |
|  |  | T2-w | 1 | 3 | Equal | 64 | GLSZM_LZHGE | | 0.6754 |
|  |  | T2-w | 1/2 | 2 | Equal | 32 | GLRLM_RLV | | 0.6734 |
|  |  | T2-w | 3/2 | 4 | Equal | 64 | GLRLM_RLV | | 0.6678 |
|  |  | T2-w | 1/2 | 2 | Lloyd | 32 | GLRLM_RLV | | 0.6673 |
|  |  | T2-w | 2/3 | 2 | Lloyd | 64 | GLSZM_GLV | | 0.6643 |
|  |  | T2-w | 1/2 | 2 | Equal | 16 | GLRLM_RLV | | 0.6631 |
|  |  | T2-w | 1/2 | 3 | Equal | 16 | GLRLM_RLV | | 0.6630 |
|  |  | T2-w | 1 | 5 | Equal | 32 | GLSZM_GLV | | 0.6618 |
|  |  | T2-w | 2/3 | 2 | Equal | 16 | GLRLM_RLV | | 0.6581 |
| **White matter** | **CET1-w** |  | 2 | 3 | Lloyd | 16 | GLCM_Correlation | | 0.7249 |
|  |  |  | 1/2 | 4 | Lloyd | 32 | Global_Skewness | | 0.7128 |
|  |  |  | 2/3 | 5 | Equal | 64 | Global_Kurtosis | | 0.7048 |
|  |  |  | 2/3 | 5 | Lloyd | 8 | Global_Kurtosis | | 0.7041 |
|  |  |  | 2/3 | 5 | Lloyd | 16 | Global_Kurtosis | | 0.7040 |
|  |  |  | 2/3 | 5 | Equal | 8 | Global_Kurtosis | | 0.7037 |
|  |  |  | 2/3 | 5 | Equal | 16 | Global_Kurtosis | | 0.7035 |
|  |  |  | 2/3 | 5 | Equal | 32 | Global_Kurtosis | | 0.7033 |
|  |  |  | 2/3 | 5 | Lloyd | 32 | Global_Kurtosis | | 0.7017 |
|  |  |  | 2/3 | 5 | Lloyd | 64 | Global_Kurtosis | | 0.7004 |
|  |  |  | 3/2 | 5 | Lloyd | 8 | GLSZM_ZSV | | 0.6951 |
|  |  |  | 1 | 4 | Lloyd | 16 | GLSZM_SZLGE | | 0.6910 |
|  |  |  | 1 | pixelW | Lloyd | 32 | GLSZM_LZE | | 0.6867 |
|  |  |  | 1 | pixelW | Lloyd | 64 | GLSZM_LZLGE | | 0.6831 |
|  |  |  | 3/2 | pixelW | Lloyd | 8 | GLSZM_LZLGE | | 0.6820 |
|  |  |  | 2 | pixelW | Equal | 8 | Global_Skewness | | 0.6769 |
|  |  |  | 2 | pixelW | Equal | 16 | Global_Skewness | | 0.6740 |
|  |  |  | 1/2 | pixelW | Equal | 8 | Global_Skewness | | 0.6713 |
|  |  |  | 2/3 | 3 | Lloyd | 8 | GLSZM_HGZE | | 0.6695 |
|  |  |  | 2 | 2 | Equal | 8 | Global_Skewness | | 0.6677 |
|  | **T2-w** |  | 2/3 | pixelW | Equal | 32 | GLRLM_LRHGE | | 0.7103 |
|  |  |  | 2 | 2 | Lloyd | 16 | Global_Variance | | 0.6976 |
|  |  |  | 2 | 2 | Equal | 64 | Global_Variance | | 0.6972 |
|  |  |  | 2 | 2 | Lloyd | 32 | Global_Variance | | 0.6969 |
|  |  |  | 2 | pixelW | Equal | 64 | NGTDM_Strength | | 0.6840 |
|  |  |  | 1 | 3 | Equal | 16 | GLSZM_ZSN | | 0.6790 |
|  |  |  | 2/3 | 2 | Lloyd | 16 | GLRLM_LGRE | | 0.6779 |
|  |  |  | 2/3 | 4 | Lloyd | 8 | GLSZM_LGZE | | 0.6779 |
|  |  |  | 2/3 | 2 | Lloyd | 32 | GLRLM_SRLGE | | 0.6739 |
|  |  |  | 1 | pixelW | Equal | 16 | NGTDM_Strength | | 0.6694 |
|  |  |  | 1 | 2 | Lloyd | 32 | GLRLM_RP | | 0.6619 |
|  |  |  | 1 | pixelW | Lloyd | 8 | GLSZM_SZE | | 0.6608 |
|  |  |  | 1 | pixelW | Equal | 32 | NGTDM_Strength | | 0.6591 |
|  |  |  | 1 | 2 | Equal | 16 | NGTDM_Strength | | 0.6546 |
|  |  |  | 2 | 3 | Equal | 64 | GLSZM_ZSN | | 0.6524 |
|  |  |  | 2/3 | 2 | Lloyd | 64 | GLRLM_LGRE | | 0.6504 |
|  |  |  | 3/2 | 1 | Equal | 16 | GLRLM_LRHGE | | 0.6503 |
|  |  |  | 3/2 | 2 | Lloyd | 16 | Global_Variance | | 0.6500 |
|  |  |  | 2 | pixelW | Lloyd | 8 | GLRLM_LGRE | | 0.6497 |
|  |  |  | 2 | pixelW | Equal | 32 | NGTDM_Strength | | 0.6493 |
|  |  |  |  |  |  |  |  |  |  |
|  | **CET1-w + T2-w** | CET1-w | 2/3 | 5 | Lloyd | 32 | Global_Kurtosis | | 0.7055 |
|  |  | CET1-w | 2/3 | 5 | Lloyd | 8 | Global_Kurtosis | | 0.7043 |
|  |  | CET1-w | 2/3 | 5 | Equal | 32 | Global_Kurtosis | | 0.7039 |
|  |  | CET1-w | 2/3 | 5 | Equal | 16 | Global_Kurtosis | | 0.7033 |
|  |  | CET1-w | 2/3 | 5 | Lloyd | 16 | Global_Kurtosis | | 0.7028 |
|  |  | CET1-w | 2/3 | 5 | Equal | 8 | Global_Kurtosis | | 0.7024 |
|  |  | CET1-w | 2/3 | 5 | Lloyd | 64 | Global_Kurtosis | | 0.7022 |
|  |  | CET1-w | 2/3 | 5 | Equal | 64 | Global_Kurtosis | | 0.7016 |
|  |  | CET1-w | 3/2 | 5 | Lloyd | 8 | GLSZM_ZSV | | 0.6969 |
|  |  | CET1-w | 1 | 4 | Lloyd | 16 | GLSZM_SZLGE | | 0.6945 |
|  |  | CET1-w | 2 | 4 | Lloyd | 8 | GLSZM_SZLGE | | 0.6783 |
|  |  | CET1-w | 1/2 | pixelW | Equal | 8 | Global_Skewness | | 0.6741 |
|  |  | CET1-w | 2 | pixelW | Equal | 16 | Global_Skewness | | 0.6740 |
|  |  | CET1-w | 2 | pixelW | Equal | 32 | Global_Skewness | | 0.6734 |
|  |  | CET1-w | 2 | pixelW | Equal | 8 | Global_Skewness | | 0.6732 |
|  |  | CET1-w | 1/2 | pixelW | Equal | 64 | Global_Skewness | | 0.6722 |
|  |  | CET1-w | 2/3 | 3 | Lloyd | 8 | GLSZM_HGZE | | 0.6722 |
|  |  | CET1-w | 1 | pixelW | Equal | 16 | Global_Skewness | | 0.6720 |
|  |  | CET1-w | 2 | pixelW | Equal | 8 | GLCM_Correlation | | 0.6710 |
|  |  | CET1-w | 2 | 2 | Equal | 8 | Global_Skewness | | 0.6682 |

**Appendix A5. Figure 1.** Comparison of AUCs between three prediction models (models 1, 2, and 3) using different combinations of radiomic features (n = 1, 5, 10, 15 and 20) in the training cohort. (a-c) model 1 using features derived from the medial temporal lobe, temporal gray matter, and temporal white matter respectively. (d-f) model 2 using features derived from the medial temporal lobe, temporal gray matter, and temporal white matter respectively. (g-i) model 3 using features derived from the medial temporal lobe, temporal gray matter, and temporal white matter respectively.


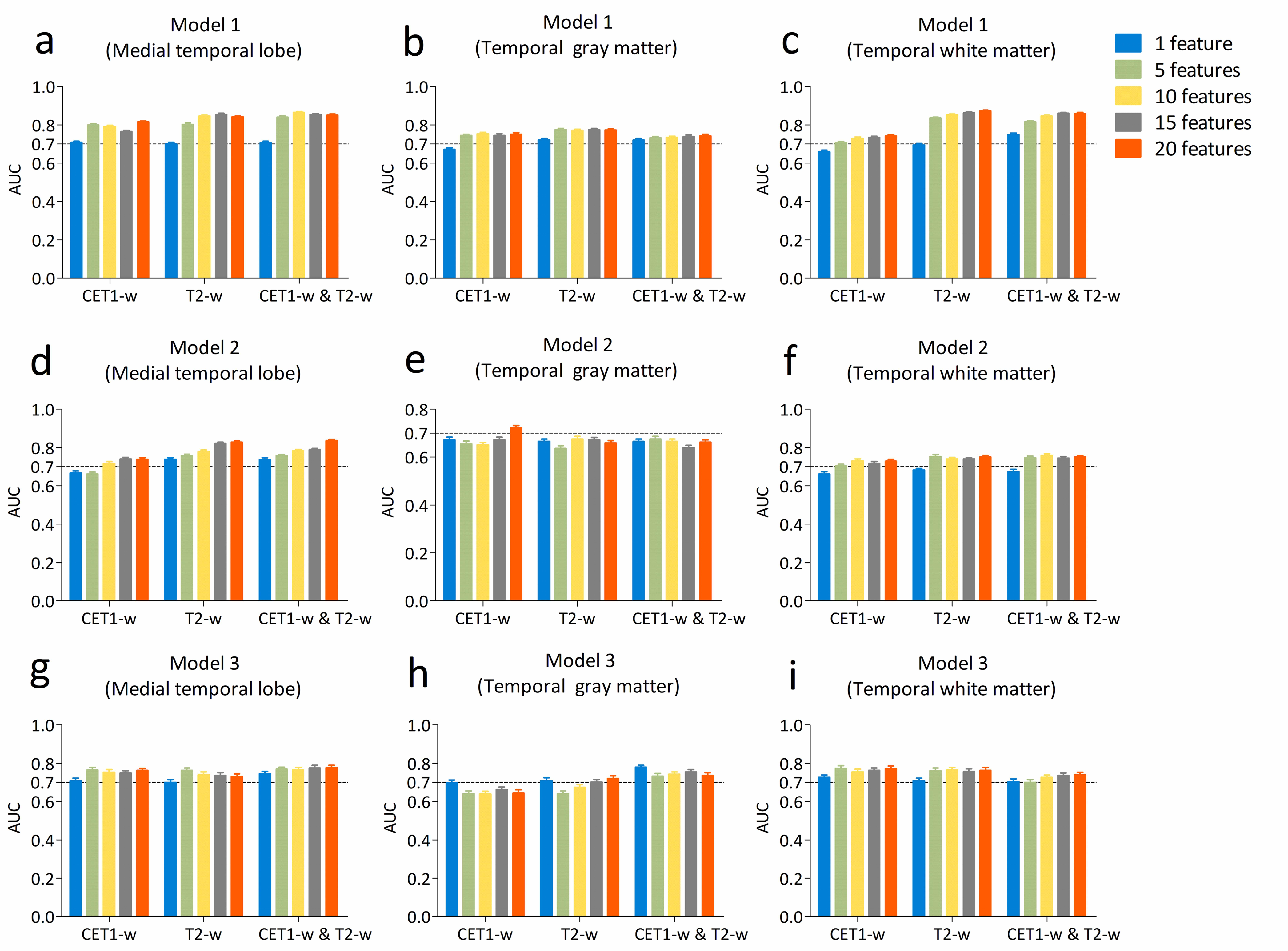

Supplement: Supplementary file 1 — Additional file 1: Appendix A1. radiomic feature extraction methodology. Appendix A2. The description of random forest method. Appendix A3. Table 1. MRI examinations of RTLI-positive and RTLI-negative patients in models 1, 2 and 3. Appendix A4. Average AUC of selected radiomic features for three models. Appendix A5. Figure 1. Comparison of AUCs between three prediction models (models 1, 2, and 3) using different combinations of radiomic features (n = 1, 5, 10, 15 and 20) in the training cohort. (a-c) model 1 using features derived from the medial temporal lobe, temporal gray matter, and temporal white matter respectively. (d-f) model 2 using features derived from the medial temporal lobe, temporal gray matter, and temporal white matter respectively. (g-i) model 3 using features derived from the medial temporal lobe, temporal gray matter, and temporal white matter respectively. [file 12885_2020_6957_MOESM1_ESM.doc]
